# Differential regulation of magnesium transporters Slc41, Cnnm and Trpm6-7 in the kidney of salmonids may represent evolutionary adaptations to high salinity environments

**DOI:** 10.1186/s12864-024-11055-x

**Published:** 2024-11-29

**Authors:** Marius Takvam, Elsa Denker, Naouel Gharbi, Valentina Tronci, Jelena Kolarevic, Tom Ole Nilsen

**Affiliations:** 1 Department of Biological Sciences, University of Bergen, Bergen, Norway; 2 NORCE, Norwegian Research Center, NORCE Environment and Climate, Bergen, Norway; 3 Faculty of Biosciences, Fisheries and Economics, The Arctic University of Norway, Tromsø, 9037 Norway

**Keywords:** Whole genome duplication, Kidney function, Magnesium transporter, Euryhaline fish, Magnesium homeostasis, Nephron tubules

## Abstract

Magnesium is important for enzymatic reactions and physiological functions, and its intracellular concentration is tightly regulated. Atlantic salmon has the ability to handle large changes in environmental Mg^2+^ concentration when migrating between freshwater and seawater habitats, making it a relevant model to investigate Mg^2+^ homeostasis. Parr-smolt transformation (PST) is a life history transition which prepares the freshwater juvenile for the marine environment. The kidney is one of the key organs involved in handling higher salt load in teleosts. Though several key Mg^2+^ transport families (SLC41, CNNM and TRPM6-7) have recently been identified in mammals and a few fishes, the molecular bases of Mg^2+^ homeostasis in salmon are not known. We found that all three families are represented in the salmon genome and exhibit a clear conservation of key functional domains and residues. Present study indicates a selective retention of paralogous Mg^2+^ transporters from the fourth whole genome duplication round (Ss4R) and a differential regulation of these genes, which suggests neo- and/or sub-functionalization events. *slc41a1-1*, *cnnm4a1*,* -4a2* and *trpm7-2* are the main upregulated genes in the kidney during PST and remain high or further increase after exposure to seawater (33 ppt). By contrast, *slc41a1-2*, *-3a*, *cnnm3-1*, and *cnnm3-2* are only upregulated after seawater exposure. In addition, *slc41a1-1*,* -2*, and *trpm7-2* respond when exposed to brackish water (12 ppt), while *cnnm3-1* and *cnnm3-2* do not, indicating the existence of a lower salinity threshold response for these members. Finally, the response of *slc41a1-1*, *-2* and *trpm7-2* in salmon was significantly reduced or completely abolished when exposed to Mg^2+^-reduced brackish water, while others were not, suggesting they might be specifically regulated by Mg^2+^. Our results are consistent with previous findings on other euryhaline teleosts and chondrichthyan species, suggesting the existence of common adaptive strategies to thrive in high salinity environments. Concomitantly, salmonid-specific innovations, such as differential regulation and recruitment of family members not previously shown to be regulated in the kidney (Cnnm1 and Cnnm4) of other vertebrates might point to adaptions associated with their very plastic anadromous life cycle.

**Supplementary Information:**

The online version contains supplementary material available at 10.1186/s12864-024-11055-x.

## Introduction

Osmoregulation is one of the key basic requirements for living cells and organisms. Aquatic animals are constantly exposed to osmotic forces across epithelial surfaces and have evolved intricate osmotic homeostatic processes involving the respiratory, digestive, and excretory systems [1–4]. In teleosts the general challenge in freshwater (FW) is passive water load and ion loss, mainly through the large surface area of the gills [1]. Fish therefore limit oral intake of water, actively absorb ions from the environment and ingested food through the gills and intestine, respectively, while producing large volumes of dilute urine through the kidney [1–3]. In seawater (SW), fish face the complete opposite challenges by passive influx of ions and osmotic water loss, and thus increase drinking rates to actively take up water through the intestine while excreting excess monovalent ions through gills [1, 5]. The kidney is primarily excreting divalent ions such as SO_4_^2-^, Mg^2+^ and Ca^2+^, producing low volumes of an isotonic urine [6, 7].

Euryhaline teleosts have evolved the ability to transit between environments with different salinities, either transiently or as part of life cycle transitions [8]. Atlantic salmon has an anadromous life cycle, where juveniles migrate from FW to a SW environment. To prepare for differences in salinity, juveniles undergo an extensive remodelling phase during a life history transition called the parr-smolt transformation (PST) [9]. Firstly, while still in FW, changes in environmental circadian cues stimulate a wave of preparatory changes in physiology, morphology, biochemistry, and behavior in the juvenile fish [10]. Following SW migration, a second set of changes provide full acclimation to the new environment [11, 12]. PST may be plastic with individuals delaying, abolishing, or reverting the process in response to adverse environmental signals [13]. The cellular and molecular mechanisms underlying tolerance to salinity transitions have started to be deciphered in the last decades, highlighting the evolutionary relevance and significance of salmonids, by providing insight into how genomic remodelling can be tied to functional diversification, plasticity, and evolutionary innovation [8, 14]. Indeed, the emergence of the salmonids is associated with a fourth round of whole genome duplication (Ss4R), which has resulted in the generation of many new gene paralogues. The study of paralogue retention of the still ongoing partial reploidization process (between 25 and 75% in salmonids [15]) has documented several cases of subfunctionalization or neofunctionalization [14, 16]. These phenomena are suspected to have provided salmonids with their remarkable plasticity in adapting to different salinity environments [17]. The molecular mechanism and ion transport in FW and SW salmonids is well studied in gills [12, 18, 19] and to a certain extent in the intestine [11, 20, 21]. However, knowledge on molecular mechanisms for ion transport in the salmonid kidney is still limited [22, 23]. Despite recent studies of salmonid-specific sulfate transporters in Atlantic salmon [24], a review clearly indicates that the mechanisms for regulation of other divalent ions, such as magnesium (Mg^2+^), in the kidney are largely unknown [3].

Controlling Mg^2+^ levels in vertebrates is vital for normal cellular, tissue, organ, and body physiology. Magnesium is essential for nucleic and protein synthesis, for intermediary metabolism and are involved in over 300 enzymatic reactions [25]. The kidney has a pivotal role in maintaining Mg^2+^ within normal physiological levels [25, 26]. Aquatic species in FW reside in low-Mg^2+^ environments (0.01–0.1 mM) where reabsorption mechanisms are vital [27–29]. On the other hand, in species inhabiting marine environments, which may contain 2500 times higher Mg^2+^ levels than FW environments, the ability to excrete excess Mg^2+^ is critical [3, 26, 30]. Teleosts have a relatively stable concentrations of Mg^2+^ in plasma, roughly 0.7–1.3 mM, irrespective of the external salinity. Thus, in euryhaline teleosts, a shift from low (FW) to high (SW) concentration of Mg^2+^ in the surrounding environment requires substantial alteration in the molecular transport pathways to switch from Mg^2+^ reabsorption to Mg^2+^ excretion through the kidney [31–33].

At the cellular and molecular level, SLC41A1-3, CNNM1-4 and TRPM6-7 have been identified as important families of transmembrane proteins involved in Mg^2+^ transport [29, 34, 35]. Both functional studies and medical data suggest that impairment of these transporters results in severe disturbances of both cellular and systemic Mg^2+^ homeostasis, and has been associated with serious diseases in both fish and mammals [36–39]. *SLC41A1*, *SLC41A3*, *CNNM2*, *TRPM6* and *TRPM7* are important for Mg^2+^ reabsorption in the kidney, and mutations in *SLC41A1*, *CNNM2*, *TRPM6* and *− 7* are underlying distinct syndromes associated with systemic hypomagnesemia [40–43]. In the stenohaline FW zebrafish, *Danio rerio*, orthologues of all these gene families, except *cnnm3*, all appear to be regulated in the kidney and be involved in reabsorption. Further, c*nnm2* and *trpm6* appear to respond to alterations in dietary Mg^2+^, and is likely involved in Mg^2+^ reabsorption in the kidney [36]. In euryhaline teleosts and chondrichthyan, some of these transporters also respond strongly to increased salinity. The Slc41a1 and Cnnm3 are proposed to be secretory Mg^2+^ transporters in the kidney [31, 32]. Recent evidence suggests that TRPM6 and − 7 not only are important for Mg^2+^ transport in mammals [34, 44, 45], but also play a role in fish [33].

To better understand the Mg^2+^ transport mechanisms the present study aimed at (1) establishing the Atlantic salmon gene repertoire for the known Mg^2+^ transporter families through data mining from the genome database followed by synteny and phylogenetic analyses, (2) investigating which of the genes are regulated transcriptionally during PST and after acclimation to full strength seawater (SW; 34 ppt) or brackish water (BW; 12 ppt), and (3) investigating the regulation of these genes when Mg^2+^ is reduced from BW using membrane nanofiltration technology.

## Materials and methods

### Experiments

#### Experiment 1: photoperiodic induction of parr-smolt transformation (PST) and seawater acclimation

Juvenile Atlantic salmon, *Salmo salar* L., (average weight 30 g) from AquaGen broodstock were obtained from the Industrial and Aquatic Laboratory, Bergen, Norway (ILAB) and randomly distributed in two experimental tanks. The experimental group (“smolt”) was kept on a 12-hour darkness and 12-hour light (12D:12 l; mimicking winter) photoperiod regime for 6 weeks, followed by 24-hours day length (mimicking summer) for 45 days at 10 °C (450 day-degrees (dd)), applying a classic photoperiodic induction of PST [46]. The control group (“parr”) remained under a constant 12D:12 l light regime during the whole experiment. Both groups were kept in 1 m^3^ 400 l rearing volume tanks supplied with flow-through freshwater (salinity; 0.01 parts per thousand (ppt), temperature; 10 ± 0.23℃, oxygen outlet water; >80%, and flow rate; 0.6 l/kg/min). The smolt group was thereafter transferred to SW (1 m^3^, 160 l rearing volume, salinity; 33 ppt, temperature; 9.2 ± 0.3℃, oxygen outlet water; <80%, and flow rate; 0.6 l/kg/min) and sampled after 1, 2 and 30 days, while the parr (control) was kept in FW during the entire experiment. Fish from both groups were fed by automatic feeders to satiation during the 12 h light phase. More details regarding ion composition in rearing water can be found in Table 1.

**Table 1:** Water quality parameters including salinity (part per million; ppt), chloride, sodium, potassium, calcium and magnesium, all calculated as mmol/L (mM) in freshwater (FW) and seawater (SW) for experiment 1, and FW, brackish water (BW) and Mg^2+^ - depleted brackish water (BW-M) for experiment 2. Note that experiment 1 was conducted in flow-through tanks and experiment 2 in recirculating aquaculture system (RAS)

Water quality	Experiment 1		Experiment 2		
	FW	SW	FW	BW	BW-M
Salinity (ppt)	0.1	32.6	0.2	11.9	11.4
Chloride (mM)	0.19	536	1.21	171.1	114.2
Sodium (mM)	0.20	426	1.57	147	98.1
Potassium (mM)	0.009	10.74	0.064	2.56	2.76
Calcium (mM)	0.027	11.23	0.249	3.56	2.37
Magnesium (mM)	0.021	49.37	0.144	16.55	9.28
Sulphate (mM)	0.018	27.27	0.022	13.8	1.076

#### Experiment 2: effect of normal and Mg2+ depleted brackish water

Juvenile Atlantic salmon smolts (start weight 140 ± 2 g from Salmo Breed, Erfjord strain), where produced in a flow-through system at the Nofima Research station in Sunndalsøra, Norway. After PST fish were distributed into three different tanks in FW (Table 1) and fish were sampled before they were redistributed into 8 octagonal tanks (2m^3^ diameter, 3.3 m^3^ volume) connected to two semi-commercial recirculating aquaculture systems (RAS) (4 tanks per system). In the first RAS, make-up FW and SW were mixed to produce brackish water (now termed BW; 12 ppt, Table 1). In the second RAS, a nanofiltration pilot plant (Fiizk, Trondheim, Norway [47]) was used to produce 12 ppt BW with reduced Mg^2+^ concentration (termed BW-M, Table 1). Fish from both BW and BW-M were sampled after 11 weeks of acclimation. Fish groups in BW and BW-M had otherwise similar tank environments (salinity of 12 ppt, oxygen outlet water < 85%, temperature; 12℃, water velocity of 1 body length per second (BL/s)). Fish were fed by automatic feeders over 24 h in excess (10% overfeeding).

Ion composition in rearing water was verified by collecting water from all experimental tanks for experiment 1 (FW and SW) and Experiment 2 (FW, BW and BW-M). All samples were analyzed for ion composition (Cl^-^; Chloride, Na^+^; Sodium, K^+^; Potassium, Ca^2+^; Calcium, Mg^2+^; Magnesium and SO_4_^2-^; Sulphate, see Table 1) by Inductively Coupled Plasma Mass Spectrometry (ICP-MS) at Norwegian Institute for Water Research (NIVA, Norway) (Experiment 1) and Norwegian University of Science and Technology (NTNU, Trondheim, Norway) (Experiment 2).

### Sampling

In both experiments, 10 individuals per group were quickly dip-netted out of the tanks and anaesthetized using a lethal dose of buffered tricaine methanesulfonate (100 mg l − 1 MS222; Sigma, St Louis, MO, USA). Blood was collected from the caudal vein and stored on ice until centrifugation (4℃, 3000 g, 5 min) and plasma aliquots were frozen. The caudal part of kidney was dissected out as described by ([24], see appendix) and preserved in RNAlater kept overnight at 4℃, then stored at − 80℃ before mRNA expression analysis. In experiment 1, gills, mid-anterior gut, hindgut, and caudal part of the kidney from juveniles in FW (*n* = 3) and SW (*n* = 3) were also sampled as described by [24, 48] and used for tissue distribution analysis.

## Analysis

### Phylogenetic and synteny analyses

Predicted sequences for Atlantic salmon (*Salmo salar*) *slc41a1-3*, *cnnm1-4* and *trpm6-7* transporters were retrieved from the Ssal_v3.1/GCA_905237065.2 genome release. Amino acid sequences from Atlantic salmon, a subset of teleost species representing the diversity of the group, as well as representative species from all other vertebrate groups, were aligned using CLUSTALW in Seaview (http://doua.prabi.fr/software/seaview). *Ciona intestinalis* and *Branchiostoma floridae* were used as outgroups. Incomplete or unpredicted sequences for Atlantic salmon, rainbow trout, elephant shark, hagfish and myxine were manually processed through BLAST and fgenesh + analyses [49] on genomic sequences, before their inclusion in the alignment (see Supplementary data). The most informative residues were selected by Gblocks (in Seaview; default parameters) and a maximum-likelihood (ML) phylogenetic analysis was run using PhyML (in Seaview; nearest neighbor interchanges and model: JTT or WAG; node support calculated using a Bootstrap analysis (100 replicates)). Phylogenetic trees were formatted using FigTree v1.4.4. To confirm the orthology and paralogy relationships of candidate genes, a synteny analysis was performed using the Genomicus online platform [50]. For salmon‐specific duplications, the pre-Ss4R configuration of Northern pike (*Esox lucius*) was used as a reference. The figures were then made using Adobe Illustrator.

### Comparative analysis of functional protein domains in Mg2+ transporters

The functional domains of SLC41A1-3, CNNM1-4 and TRPM6-7 previously characterized or predicted in mammals (from the literature) as well as pathogenic mutations identified in patients (literature and ClinVar, OMIM and UniProt variant databases) were mapped onto the corresponding amino acid alignment to analyze sequence conservation across vertebrates. Residue conservation was displayed using Boxshade (threshold = 0.5). Putative phosphorylation sites were predicted using NetPhos3.1 (https://services.healthtech.dtu.dk/services/NetPhos-3.1/*).*

### Isolation of total RNA and first strand cDNA synthesis

Approximately 20–25 mg of gill and kidney tissue were homogenized in 600 µl of RLT plus buffer and Reagent DX (Qiagen QIAsymphony total RNA extraction kit, Hilden, Germany) using stainless steel beads (3 mm, Qiagen, Germany) and the Precellys 24 tissue homogenizer (Bertin Technologies, Montigny-le‐Bretonneux, France). Total RNA was extracted using a QIAsymphony Robot (Qiagen, Germany), following the manufacturer’s protocol (Qiagen, Germany). Roughly 25–30 mg of anterior gut and hindgut was isolated using the TRI Reagent method according to Chomczynski [51]. Total RNA from all tissues were eluted in 100 µl ultrapure water and RNA concentrations quantified using an Invitrogen Qubit 4 Fluorometer (Thermo Fisher Scientific, USA) applying the QubitTM RNA HS Assay Kit protocol (InvitrogenTM, Thermo Fisher Scientific, USA). Integrity of total RNA was validated using Agilent RNA 6000 Nano kit in an Agilent 2100 expert analyzer (Agilent technologies, USA). cDNA was synthesized using 1000 ng total RNA and Oligo (dT) 20 primer in conjunction with SuperScript™ III Reverse Transcriptase kit (Invitrogen, USA) according to the manufacturer’s instructions.

#### Tissue distribution and temporal gene expression profiles using real-time qPCR

Real-time quantitative PCR (qPCR) was carried out using iTaq™ Universal SYBR^®^ Green Supermix (Bio-Rad Laboratories, USA) in a total volume of 12.5 µl, using exon junction‐spanning primers (Table 2) at final concentration of 200 nM. The reactions were run in a C1000 Touch™ Thermo cycler, CFX96™ Real‐Time PCR detection System and CFX Manager software (software version 3.1; Bio‐Rad Laboratories). The thermal conditions consisted of an initial denaturation for 2 min at 95 °C, followed by 37 cycles at 95 °C for 15s and 60 °C for 25s. Melt curve analysis verified that the primer sets for each qPCR assay had no primer dimer artefacts and generated a single product. Only primers with an amplification efficiency > 80% (Table 2) were selected and the threshold value for validated gene expression was set at 30 [52]. cDNA dilution used was 1:10. Validation of the endogenous reference gene(s) glyceraldehyde-3-phosphate dehydrogenase (*gapdh)*, elongation factor 1 alpha (*ef1a)* and actin (*β*‐*actin)* were conducted using RefFinder [53], which includes BestKeeper [54], NormFinder [55], Genorm [56] and the comparative delta Ct method [57]. *ef1a* was determined as the most stable reference gene for normalization. Relative expression was calculated according to the PCR efficiency-corrected formulas from Pfaffl [54].

**Table 2:** Primer sequences and GenBank accession numbers designed primers

Gene	Primer forward (5´- 3`)	Primer reverse (5´- 3`)	Accession number
*slc41a1-1*	GGACATCGTTCAGCACTGGA	AGGCCAATGTCATCTCCAGG	XM_014132936.2
*slc41a1-2*	GGTGTTTACTGAGGTGACGG	TGACCAATATTAGCCGCTGTG	XM_014166950.2
*slc41a2a*	GTGTTCTTGGGCTGGGTACT	ACCATGATGATCCCCTGCAG	XM_014169899.2
*slc41a2b1*	CAAGAAGACGGGCATCAACC	CATACGGGTGAGAGTCCAGG	XM_014208073.2
*slc41a2b2*	GCTTCATCATGGTGGGAGTG	GTGAGAGTCCAGGCAGTTGT	XM_045698502.1
*slc41a3a*	ACGGTGGTGGGCTTTCTG	ACCCCAATCATTACCAGACCTA	XM_014135697.2
*cnnm1a2*	AGATTGTCTGGAAGGCCATCC	CGACGATGACTGCTCCATGC	XM_014180091.2
*cnnm1b1*	CGAGACTGACCTCTACACTGAT	TGTGGACAGGAAGCGGTG	XM_045708923.1
*cnnm2b1/2*	GCAGTACATCAAGGTGACCC	GTGTGTTGAGGTTGAGCAGG	XM_014177888.2/ XM_045691172.1
*cnnm3-1*	AGACCCTTCTGAAATCAAGATCCTG	CATGTGACTGTCGGTTGGTCG	XM_014128009.2
*cnnm3-2*	CAAGTTCTACAATCACCCACTG	TTCATGTCCATGTAGCCGTCTG	XM_014140511.2
*cnnm4a1*	CCCGTCCCAGATATCGGAC	TCCGTAGTAGGAGAAGGGTCC	XM_045695316.1
*cnnm4a2*	TCCCACGTTAGAGTTCTGCT	GGTGTATGTTGGTGAGGGGA	XM_014170484.2
*cnnm4b1*	TGAGTCCGACCTTTATACTGACA	TTCACCTTGCACTCGTTCTC	XM_014128014.2
*cnnm4b2*	TCGCTTCCCAGAGTTCCG	AAGTCTGGTGTGTACTGGCA	XM_014140190.2
*trmp6-1*	ACGGTTCATGGAGGGACAG	TTCCTGAGGTCATGGGTGC	XM_014172215.2
*trmp6-2*	GAATGCAGCTCCTACAATGTC	CTCTTCTTGCACTGCCTGG	XM_045702993.1
*trmp7-1*	GCTCAAAGACGTGGTCTTC	CACATTCGTATCAGTGGCAC	XM_045708037.1
*trmp7-2*	CAAAGACAGAACCTCTATTTCCAC	GGCTGAGCTGTCTGGAGG	XM_045689351.1
*ef1a*	CCCTGTGGAAGTGGCTGAAG	CATCCAAGGGTCCGTATCTCTT	Olsvik et al., [58]
*gapdh*	AAGTGAAGCAGGAGGGTGGAA	CAGCCTCACCCCATTTGATG	Olsvik et al., [58]
*β-actin*	CCAAAGCCAACAGGGAGAAG	AGGGACAACACTGCCTGGAT	Olsvik et al., [58]

### Plasma analyses

A Pentra c400 clinical chemistry analyzer with ISE module (HORIBA, Kyoto prefecture, Japan) was used for determining ion concentration in plasma. To measure Na^+^ and Cl^-^, the Ion Selective Electrode (ISE) module was used while the ABX Pentra Magnesium RTU reagent (HORIBA) was used for the quantitative in vitro diagnostic determination of Mg^2+^. Calibration of all reagents was performed using the ABX Pentra Multical and ABX Pentra N and P control following manufacturer’s protocol. The ISE module was calibrated using the ABX Pentra Standard 1, ABX Pentra Standard 2 and ABX Pentra Reference.

### Statistical analysis

Statistical differences were determined either by linear models (One-Way or Two-way ANOVA) followed by a Tukey’s HSD post-hoc test. To determine distribution, normality (Q-Q- plots), homogeneity of variance (scale location plots) and influential outliers (residuals vs. leverage with Cook’s distance) was performed on all datasets. A mix model analysis was performed to exclude potential tank effects during the experiments. Results are presented as mean ± the standard error of mean (SEM) and considered significant at *P* < 0.05 level.

## Results

### The Slc41 family in Atlantic salmon

**Fig. 1:**
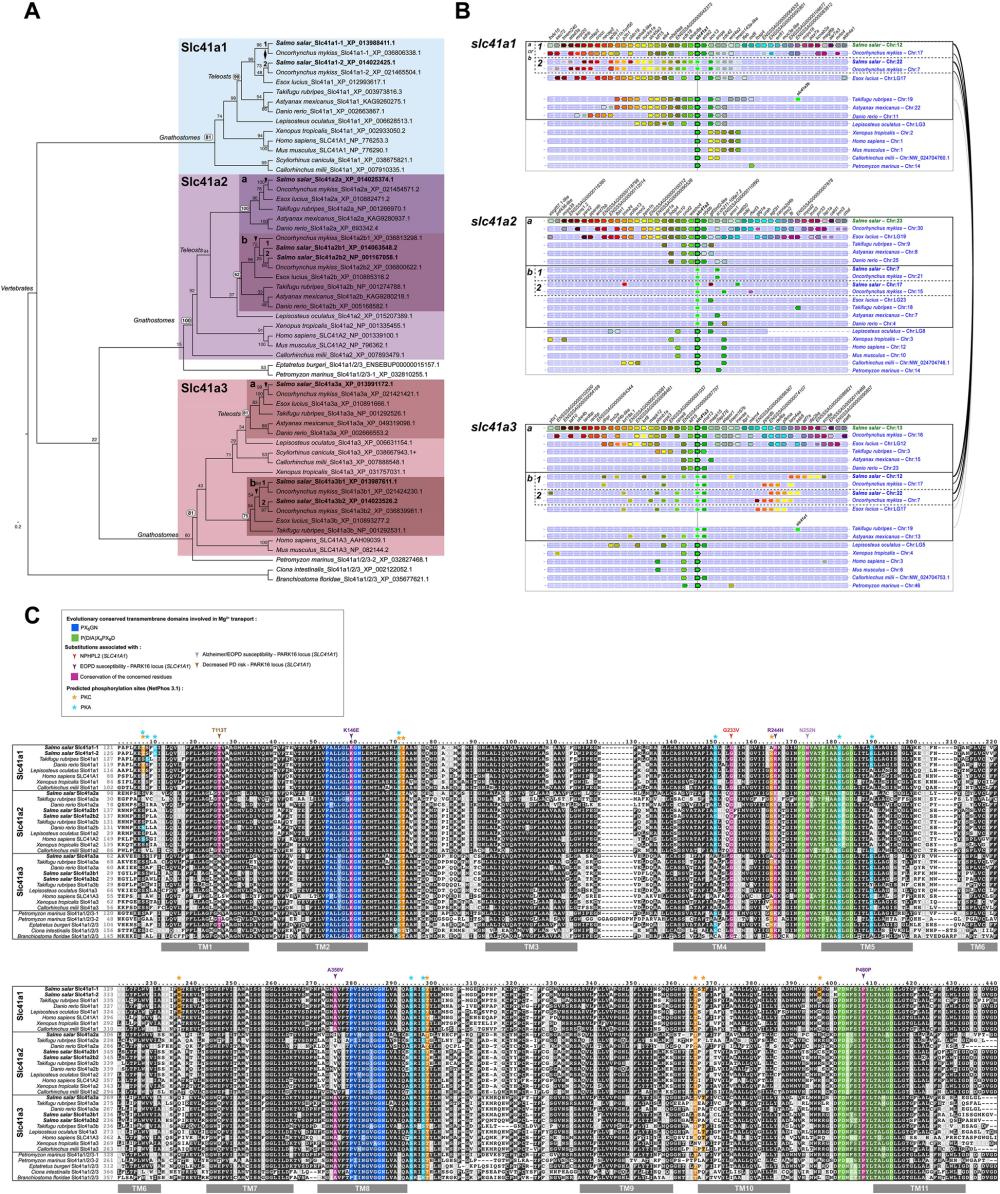
Atlantic salmon gene repertoire for the Slc41 magnesium transporter family.** A **Phylogenetic reconstruction showing the relationship between vertebrate Slc41a1, -a2, and -a3 subfamilies and the position of Atlantic salmon sequences (bold) within those. Maximum likelihood reconstruction on aminoacid sequences using WAG with 4 rate classes and bootstrap values calculated for 100 replicates; *C. intestinalis* and *(B) floridae* sequences are used as outgroup. **B **Synteny analysis confirmed orthologous and paralogous relationships between the *slc41a1*, -*a2*, and -*a3* genes inferred from the phylogenetic analysis, using Genomicus. Genes with the same fill color are homologous and among them, the ones with a black stroke are orthologous to one another across species, and within a species the ones presenting a white stroke are both paralogous to one another and the ones with a black stroke. The thick-lined boxes group orthologues resulting from teleost-specific duplications and the dashed-lined boxes the ones resulting from salmonid-specific duplications. The brackets on the right side connect gene landscapes on the same chromosome. **C **Aminoacid sequence alignment displaying residue conservation across vertebrate sequences, using Boxshade (threshold = 0.5). The colour boxes highlight sequence conservation for specific domains of residues proposed or known to hold key structural or functional properties for the protein, and/or associated with known pathogenic mutations (see legend box for details). TM1-11 indicates predicted transmembrane domains

The SLC41 family consists of three members in chondrichthyans, sarcopterygians and non-teleost actinopterygians, namely SLC41A1, −2 and − 3. After the third round of whole genome duplication occurring in teleosts (Ts3R), up to five genes are found, namely *slc41a1*, *slc41a2a*, *slc41a2b*, *slc41a3a*, and *slc41a3b*, indicating retention of both Ts3R duplicates for *slc41a2* and *slc41a3* but the loss of one for *slc41a1* (Fig. 1a, b). Within teleosts, salmonids have undergone a fourth round of whole genome duplication (Ss4R), and we found that in Atlantic salmon and rainbow trout, all five Ts3R paralogues were retained, with *slc41a1*, *slc41a2b* and *slc41a3b* being represented by two Ss4R paralogues each, while *slc41a2a* and *slc41a3a* have both retained a single Ss4R paralogue. Thus, the Slc41 repertoire consists of *slc41a1-1*, *1–2*, *−2a*, *−2b1*, *−2b2*, *−3a*, *−3b1*, and − *3b2* (Fig. 1a, b).

The two PX6GN and the two P(D/A)X4PX6D motifs, known to be the evolutionary conserved transmembrane domains involved in Mg^2+^ transport in the SLC41 family [59, 60], were 100% conserved for all the species in our dataset, including the two salmonid species (Fig. 1c). We then analyzed the conservation of seven amino acids known to be involved in pathogenic substitutions in humans, located on conserved regions within vertebrates, and most of them located within or in close proximity of these motifs (arrowheads on Fig. 1c). G233 in human SLC41A1, involved in NPHPL2 (nephronophthisis-like nephropathy, an autosomal recessive early onset renal insufficiency) [38], was conserved in all salmon Slc41 family members, and more largely in all the proteins in the dataset (Fig. 1c). SLC41A1 is also associated with the PARK16 locus, involved in susceptibility for early onset Parkinson disease (EOPD) and/or for Alzheimer’s disease, and we found the amino acids T113 [61, 62], R244 [63], N252 (ClinVar), A350 [64, 65] and P480 [66] in human SLC41A1 also to be conserved in salmon and all other vertebrates in our alignment. R244, N252 and P480 were conserved within all three Slc41 members, while T113 was conserved in Slc41a1 and − 2, and A350 in Slc41a1 and − 3 (Fig. 1c).

### The Cnnm family in Atlantic salmon

**Fig. 2:**
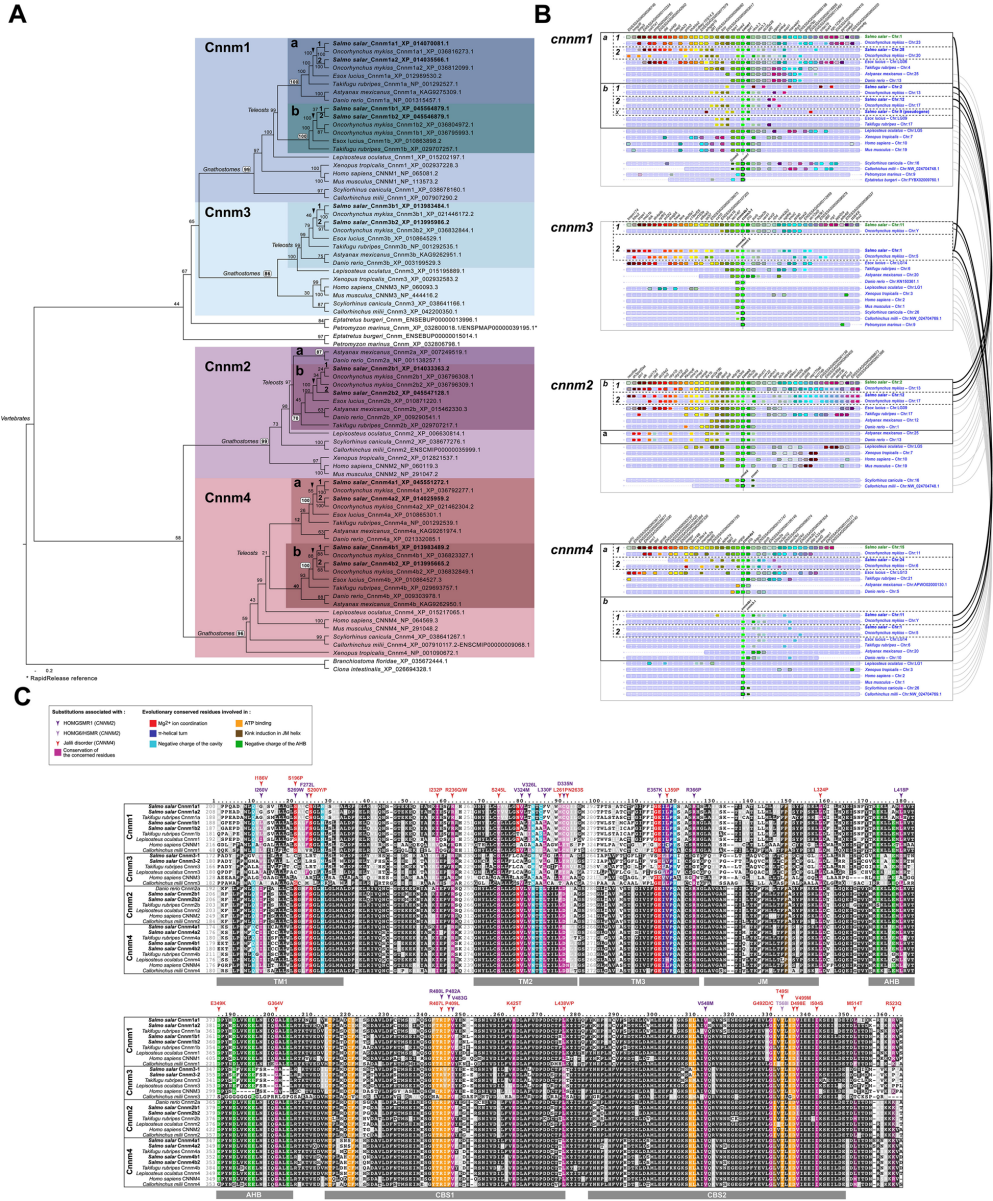
Atlantic salmon gene repertoire for the Cnnm magnesium transporter family. **A** Phylogenetic reconstruction showing the relationship between vertebrate Cnnm1, −2, −3, and − 4 subfamilies and the position of Atlantic salmon sequences (bold) within those. Maximum likelihood reconstruction on aminoacid sequences using JTT with 4 rate classes and bootstrap values calculated for 100 replicates; *C. intestinalis* and (*B*)* floridae* sequences are used as outgroup. **B** Synteny analysis confirmed orthologous and paralogous relationships between the *Cnnm1*, -*2*, *−3*, and − *4* genes inferred from the phylogenetic analysis, using Genomicus. Genes with the same fill color are homologous and among them, the ones with a black stroke are orthologous to one another across species, and within a species the ones presenting a white stroke are paralogous to one another and to the ones with a black stroke. The thick-lined boxes group orthologues resulting from teleost-specific duplications and the dashed-lined boxes the ones resulting from salmonid-specific duplications. The brackets on the right side connect gene landscapes on the same chromosome. **C** Aminoacid sequence alignment displaying residue conservation across vertebrate sequences, using Boxshade (threshold = 0.5). The colour boxes highlight sequence conservation for specific domains of residues proposed or known to hold key structural or functional properties for the protein, and/or associated with known pathogenic mutations (see legend box for details). TM1-3 are predicted transmembrane domains; JM = juxtamembrane helix; AHB = acidic helical bundle; CBS1-2 = cystathionine-β-synthase domains

In chondrichthyans, sarcopterygians and non-teleost actinopterygians, the CNNM family comprises 4 members, CNNM1, 2, −3, and − 4 (Fig. 2a, b). *CNNM2* and − *4* appear to originate from a common ancestor, while *CNNM1* and *− 3* appear more closely related (Fig. 2a). In these groups, *CNNM3* and *− 4* are immediate neighbors, and *CNNM1* and *− 2* are on the same chromosome, and only one gene apart in chondrichthyans (Fig. 2b), suggesting they result from the duplication of an ancestral pair of neighbor genes, *CNNM1/3* and *CNNM2/4*. As a result of Ts3R, up to seven genes are found in teleosts (including Northern pike), namely *cnnm1a*, *−1b*, *−2a*, *−2b*, *−3*, *−4a*, and *− 4b*, indicating a loss of one of the cnnm3 Ts3R duplicates (Fig. 2a, b). The chromosome linkage was maintained, with the -a and the -b paralogues of the 1–2 and 3–4 linked pairs grouping together (Fig. 2b). *cnnm3* is on the same chromosome as *cnnm4b*, indicating that the teleost *cnnm3* is the -b paralogue, and that it is *cnnm3a* that was lost. Euteleostei, including the Northern pike, have in addition lost *cnnm2a*, and Atlantic salmon presents two Ss4R paralogs of each of the six retained genes, namely *cnnm1a1*, *−1a2*,* −1b1*,* −1b2*,* −2b1*,* −2b2*,* −3(b)1*,* −3(b)2*,* −4a1*,* −4a2*,* −4b1*, and *− 4b2* (Fig. 2a, b). The chromosome linkage was preserved in salmon, at the level of *−1* and *− 2* paralogues. Interestingly, an Atlantic salmon-specific chromosome rearrangement brought *cnnm1a1* on the same chromosome as *cnnm3(b)2* and *cnnm4b2*.

All the five residues functionally shown to be key for Mg^2+^ ion coordination in the CNNM family (red background in Fig. 2c [67]), were conserved in the salmonid Cnnm3 paralogues, except for Cnnm3-1 and − 2. These proteins, as for most vertebrate CNNM3, only retained one out of five. All the fourteen sites involved in ATP binding were fully conserved (orange background in Fig. 2c [67]), except for one, specifically in salmonids, again in Cnnm3-1 and − 2. The other fifteen residues key for the general protein structure, including the negative charges in the transmembrane pockets (light and dark blue, green, and brown background in Fig. 2c [67]), were all conserved, except seven of them in CNNM3 of all vertebrate species, with one of the positions being also specifically not conserved in salmon Cnnm1b1 and − 2. Finally, we analyzed the residues involved in pathogenic substitutions in humans and leading to HOMGSMR1 (Hypomagnesemia, Seizures, and Impaired Intellectual Development 1 [43, 68–72], unpublished ClinVar submissions [73]) or HOMG6/HSMR (Hypomagnesemia 6, Renal/Hypomagnesemia, Seizures, and intellectual disability [74]) when present in CNNM2, and Jalili disorder (retinal cone-rod dystrophy and amelogenesis imperfecta [75–90], and unpublished ClinVar submissions [91]) when present in CNNM4. Twelve of them were located on the functionally characterized residues described above, while the 29 others were not, but were often located in proximity of them (arrowheads on Fig. 2c). The above residues were well conserved in vertebrates, including salmonids, except for S196 (human CNNM4)/S269 (human CNNM2), V324 and 326 in CNNM2, and M514 and R523 in human CNNM4, which are largely not conserved in vertebrate CNNM3 sequences. Interestingly, I186 on human CNNM4, I260 on human CNNM2 as well as F272, V326 and L330 on human CNNM2 were not conserved in the clade comprising salmonid Cnnm1a1 and − 2 and fugu Cnnm1a. F272 and V326 were generally not conserved in CNNM3 sequences.

### The TRPM1/3/6/7 subfamily in Atlantic salmon

**Fig. 3:**
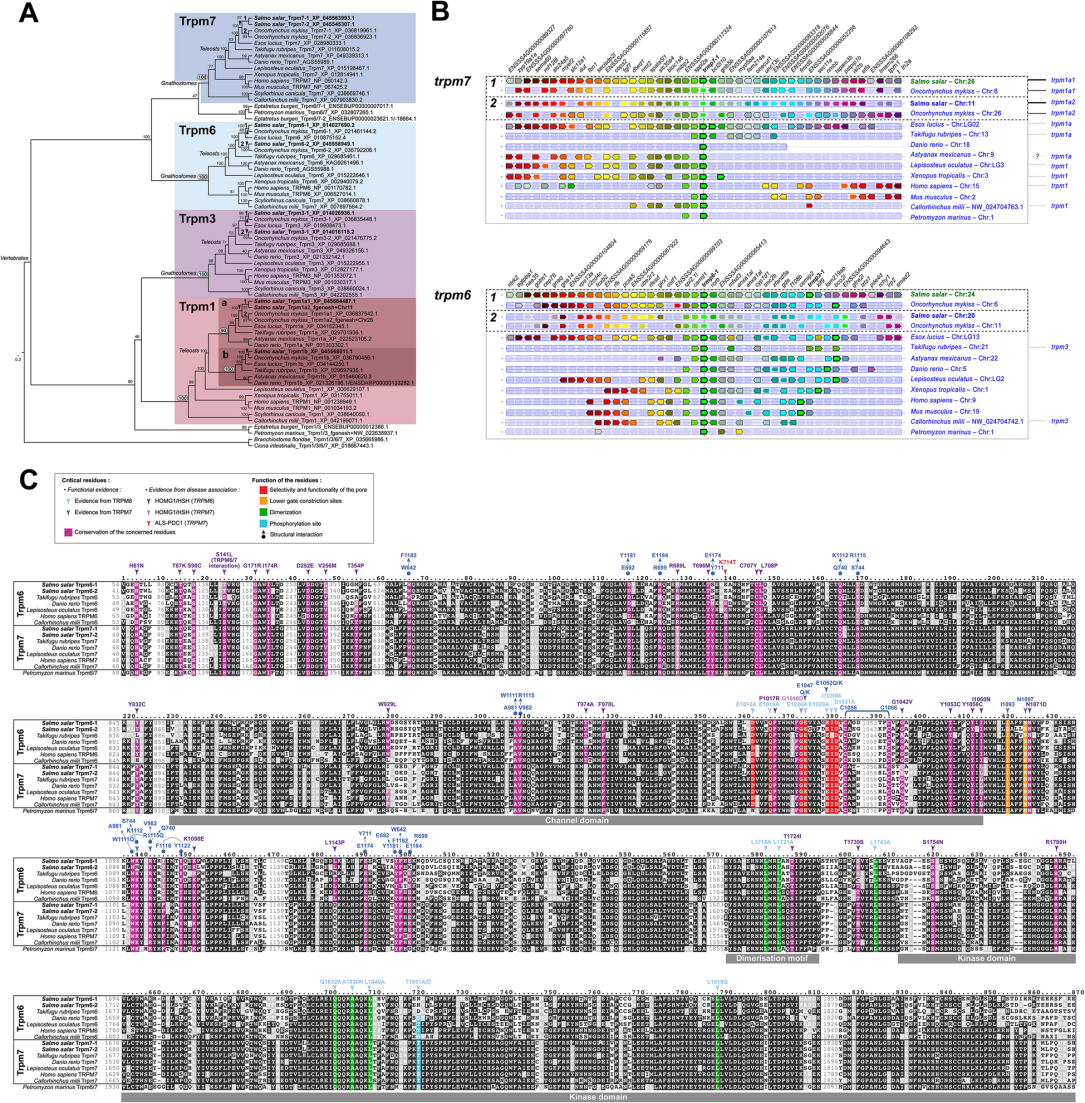
Atlantic salmon gene repertoire for the Trpm 6/7 magnesium transporter subfamily. **A** Phylogenetic reconstruction showing the relationship between vertebrate Trpm1, −3, −6, and − 7 subfamilies and the position of Atlantic salmon sequences (bold) within those. Maximum likelihood reconstruction on aminoacid sequences using JTT with 4 rate classes and bootstrap values calculated for 100 replicates; *C. intestinalis* and (*B*)* floridae* sequences were used as outgroup. **B** Synteny analysis confirming the orthologous and paralogous relationships between the *Trpm6* and − *7* genes inferred from the phylogenetic analysis, using Genomicus. Genes with the same fill color are homologous and among them, the ones with a black stroke are orthologous to one another across species, and within a species the ones presenting a white stroke are paralogous to one another and to the ones with a black stroke. Dashed-lined boxes are the ones resulting from salmonid-specific duplications. The brackets on the right side connect gene landscapes on the same chromosome. **C** Amino acid sequence alignment displaying residue conservation across vertebrate sequences, using Boxshade (threshold = 0.5). The colour boxes highlight sequence conservation for specific domains of residues proposed or known to hold key structural or functional properties for the protein, and/or associated with known pathogenic mutations (see legend box for details)

Though only TRPM6 and − 7 are known to be involved in Mg^2+^ transport, we included TRPM1 and − 3 in our analyses to increase confidence in our inferences and obtain a full picture of the evolution of the subfamily. In chondrichthyans, sarcopterygians and non-teleost actinopterygians, the TRPM1/3/6/7 subfamily members are each present in one copy (Fig. 3a, b). *TRPM6* and *− 7* appears to originate from a common ancestor, while *TRPM1* and *− 3* appears more closely related (Fig. 3a). In addition, we observed that in these groups, *TRPM1* and *− 7* were located on the same chromosome, while *TRPM3* and *− 6* are located on the same chromosome (Fig. 3b), suggesting they result from the duplication of one original pair of linked genes, *TRPM1/3* and *TRPM6/7*. Only five genes were found in teleosts (including Northern pike) after Ts3R, namely *trpm1a*, *−1b*, *−3*, *−6*, and *− 7*, indicating a loss of one of the Ts3R duplicates for every family member, except for *trpm1* (Fig. 3a, b). Chromosome linkage was maintained between *trpm1a* and *− 7* (indicating that the kept *trpm7* paralogue is *trpm7a*), and *trpm3* and *− 6* (Fig. 3b). In salmonids this family consists of *trpm1a1*,* −1a2*,* −1b*,* −3-1*,* −3-2*,* −6-1*,* −6-2*,* −7-1*, and *− 7 − 2*, indicating that all Ss4R paralogues were retained, except for *trpm1b*. The chromosomal linkage was kept at the level of *−1* and *− 2* paralogues.

All the residues known from functional studies to be essential for the Mg^2+^ selectivity and the functionality of the pore in TRPM6 and − 7 were found to be conserved (red and orange back-ground in Fig. 3c, [92–94]). Interestingly, the I1030 residue was not conserved in the fugu. The three leucines shown to be important for dimerization in human TRPM6 were also fully conserved (green background in Fig. 3c, [95]). T1851 has been shown to be an important phosphorylation site in TRPM6, however, this was not conserved in salmon Trpm6-1 and − 2 and in fugu Trpm6, while it was conserved in the Trpm7 orthologues in both species (light blue background in Fig. 3c, [96]). Out of the 19 sites in human TRPM7 shown to be involved in 10 pairwise structural interactions, 17 were conserved in salmon except for K1112 and E1184 in human TRPM7, which are not conserved in salmon Trpm6-2 (dark blue arrows in Fig. 3c, [93, 97]). TRPM6 and − 7 are both involved in the human disease HOMG1 (intestinal hypomagnesemia with secondary hypocalcemia). Analysis of 29 residues known to be mutated in TRPM6 and one in TRPM7 (dark and light purple arrowheads on Fig. 3c) shows that out of these 29 positions, only one was not conserved in salmon, namely T354 present in human TRPM6, which neither was conserved in Trpm6-1 or Trpm6-2. Interestingly, this position was again not conserved in fugu Trpm6. Two other positions, Y832 and L1143, were conserved in all species except for fugu Trpm6. It is worth noting that among the conserved positions, S141 in human TRPM6 is known to be involved in TRPM6/TRPM7 interaction. TRPM7 is also involved in susceptibility to ALS-PDC (Amyotrophic lateral sclerosis-parkinsonism/dementia complex of Guam), and the residue mutated in human TRPM7 (red arrowhead on Fig. 3c) is conserved in the vertebrate TRPM6 and − 7, except for in salmon Trpm6-1 and 6 − 2 and fugu Trpm6.

### Tissue distribution analysis of Mg2+ transporter repertoire in gills, gut and kidney

**Fig. 4:**
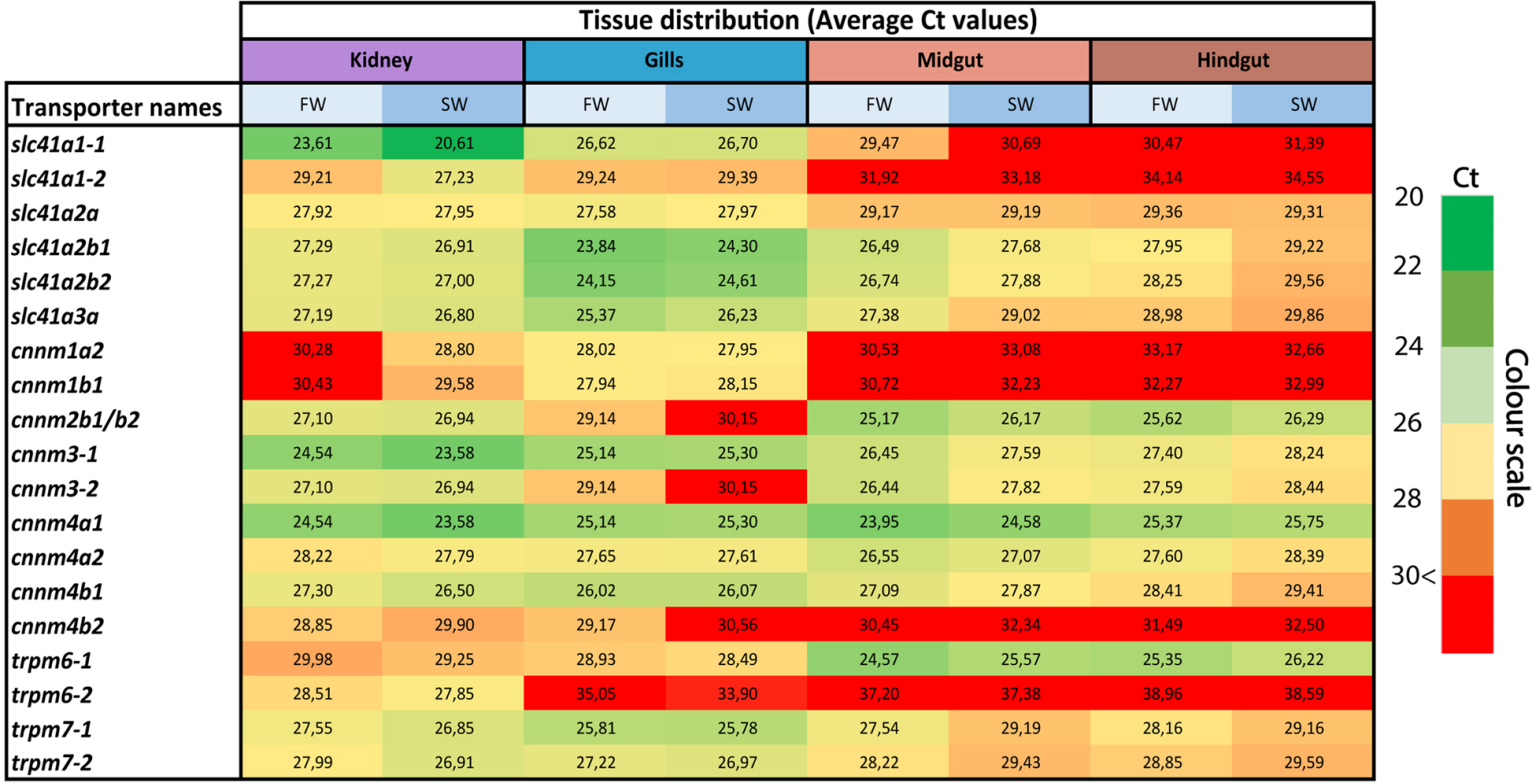
Heatmap visualization of RT-qPCR analysis of mRNA levels for all the identified *slc41a1-3*, *cnnm1-4* and *trpm6-7*family members in the kidney, gill, midgut and hindgut of Atlantic salmon in freshwater (0.1 ppt) and seawater (33 ppt) environments. This heatmap illustrates trends in tissue distribution and environmental-specific variations in threshold (Ct) values for the diverse gene candidates, as well as differences between Ss4R paralogues, through color gradients spanning from green (high expression) to red (no expression). Generally, there is higher variability when Ct values exceeds 30 [52] and therefore a clear cut has been made for values > 30 for this dataset. Data is presented as mean values for each tissue in both freshwater (*n* = 3) and seawater (*n* = 3)

The candidate genes identified in our phylogenetic analysis were analyzed for tissue distribution in the three main osmoregulatory organs in Atlantic salmon, namely the gills, gut (divided into midgut and hindgut) and kidneys, both in FW and SW acclimated salmon (Fig. 4; Ct values). We found all the putative Mg^2+^ transporters were expressed in the kidney, with *slc41a1-1*, *cnnm3-1* and *cnnm4a1* having the highest expression. While *trpm6-2* appeared exclusively expressed in the kidneys, the others were also found in the gills (*slc41a1-1*, *−2*, *cnnm1a2*,* −1b*, and *− 4b*) or both the gills and the intestine (*cnnm2b1/2* and *− 3 − 2*) (Fig. 4). These results indicate clear tissue-specific differences between the various Mg^2+^ transporters, including between salmon-specific paralogues. Further, *slc41a1-1*, *−2*, *cnnm1a2*, *−3-1*, *−4a1*, *trpm6-2* and *− 7 − 2* displayed a higher expression in salmon acclimated to SW compared with their FW counterparts (Fig. 4), suggesting an environmental regulation of these genes.

### Expression of Mg2+ transporters in the kidney during photoperiodic induction of parr-smolt transformation (PST)

**Fig. 5:**
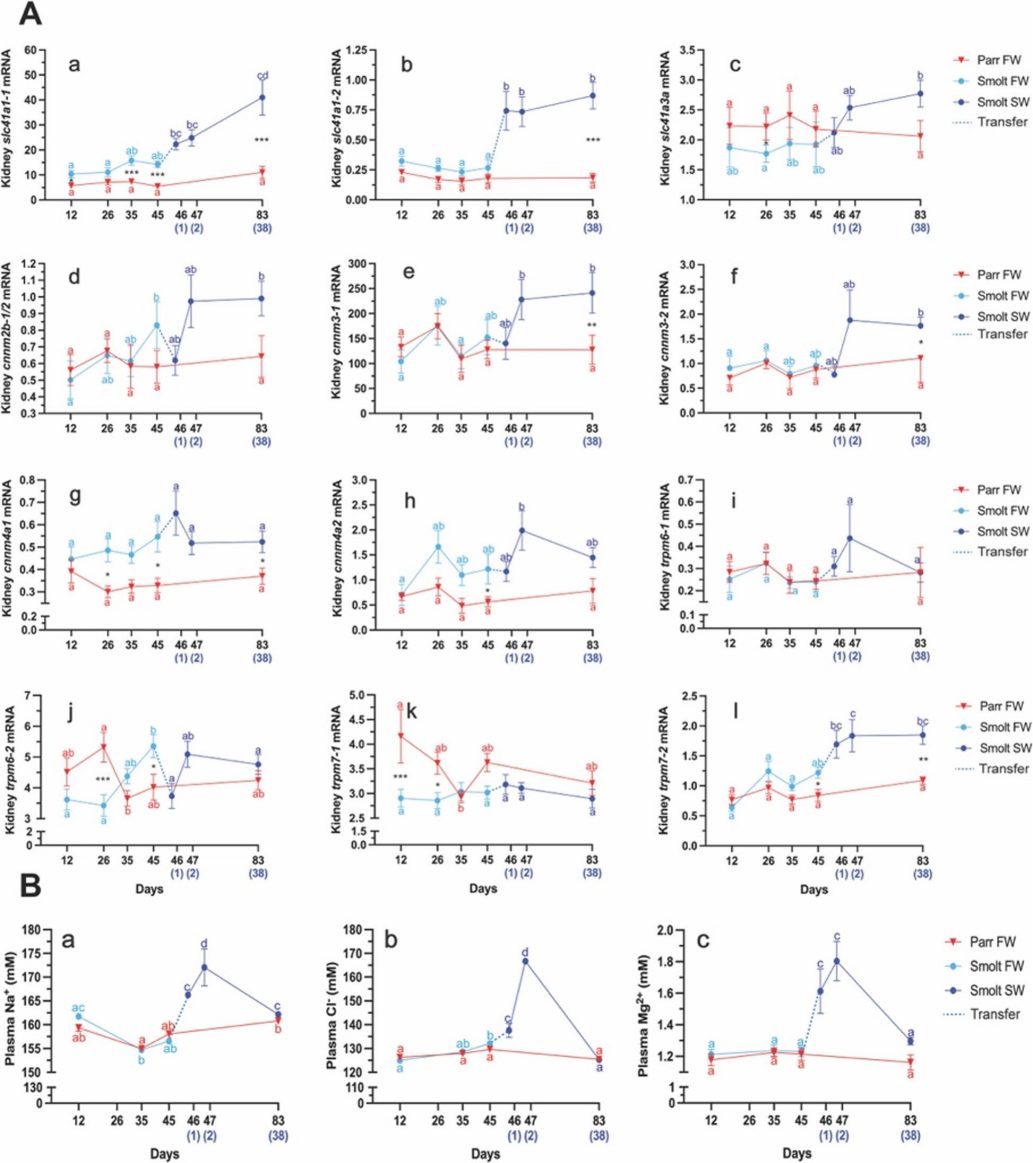
Relative gene expression levels and plasma ion concentrations in the kidney of Atlantic salmon during PST (FW; 0.01 ppt) and seawater (SW; 33 ppt) acclimation. **A** Relative expression levels of **a**: *slc41a1-1*, **b**: *slc41a1-2*, **c**: *slc41a3a*, **d**: *cnnm2b-1/2*, **e**: *cnnm3-1*, **f**: *cnnm3-2*, **g**: *cnnm4a1*, **h**: *cnnm4a2*, **i**: *trpm6-1*, j: *trpm6-2*, **k**: *trpm7-1* and **l**: *trpm7-2*. **B** Ion concentrations (mM) in plasma during PST in freshwater (FW: 0.01 ppt) and after seawater transfer (SW: 33 ppt), **a**: sodium (Na^+^) **b**: Chloride (Cl^-^) and **c**: magnesium (Mg^2+^). Different letters denote significant effects within the parr (red) and smolt (FW: light blue, SW: dark blue) groups, reflecting the interplay of photoperiod and salinity across time (days). Asterisks (*) denote significant difference between parr and smolt groups throughout the FW (photoperiod) and SW (seawater acclimation) phases, and significance levels are indicated as *P < 0.05, **P < 0.01, and ***P < 0.0001. Note that the parr group (control) remains in freshwater throughout the experimental period. Data is presented as mean ± sem (*n* = 8–10)

We first compared the relative expression of our candidate genes in FW, with or without induction of PST (light blue vs. red on Fig. 5) to investigate the effect of photoperiod driven changes. *slc41a1-1* were persistently higher expressed in the kidney during PST in the smolt group compared with the control parr group at 12d, 35d and 45d. (Fig. 5A; a). Similarly, *cnnm4a1* were significantly higher expressed in smolts than parr at 26d and 45d (Fig. 5A; g), while *cnnm4a2* and *trpm7-2* were only significant at the last time point (45d, Fig. 5A; h and l). By contrast to *slc41a1-1*, *slc41a1-2* did not present any significant difference between groups during PST, just a slight trend to higher expression in the smolt group. While *slc41a1-1*, *cnnm4a1* and *− 2*, and *trpm7-2* were significantly higher expressed in smolts than parr, despite no significant increase between time points, *cnnm2b1/2* presented a significant increase between the 12d and 45d, but no significant difference with the expression in the parr group (Fig. 5A; d).

Interestingly, *slc41a3a*, *trpm6-2*, and *trpm7-1* were significantly lower expressed in smolts during PST than the control parr group, particularly at 26d (Fig. 5A; c, j, k). In the case of *slc41a3a*, the trend was consistent throughout the whole period, despite large variation within groups. In the case of *trpm6-2* and *trpm7-1*, difference in relative expression arose from a significant decrease in the parr group, while *trpm6-2* displayed an increasing trend (Fig. 5A; j), and expression of *trpm7-1* remained stable (Fig. 5A; l). As a consequence, *trpm6-2* expression in smolts were higher than in parr at 45d (Fig. 5A; j) whereas *trpm7-1* was significantly lower in smolts at 12d (Fig. 5A; l). Relative expression of *trpm6-1* and *trpm6-2* did not differ significantly between groups during PST.

### Effect of seawater transfer on Mg2+ transporter expression in smolts

Groups that underwent PST was transferred to full-strength seawater (33 ppt) (dark blue vs. red on Fig. 5). We observed that the salinity resulted in a significant increase of *slc41a1-1*, *slc41a1-2*, *slc41a3a*, *cnnm3-1*, *cnnm3-2*, cnnm4a2, and *trpm7-2* compared to expression levels in FW smolts (Fig. 5A; a,b, c,e, f,g, l). The expression of *slc41a1-1*, *slc41a1-2*, and *trpm7-2* were already significantly elevated after 1 day in SW (Fig. 5A; b, c, l), while *cnnm3-1* and *cnnm4a2* expression increased after two days for (Fig. 5A; e, h) Expression of *slc41a3a* and *cnnm3-2* only became significant after 38 days in SW (Fig. 5A; c, f). By contrast, the SW transfer led to a significant decrease in the expression of *trpm6-2* (Fig. 5A; g). If we now compare to the parr group at day 83, all these genes had a significantly higher expression in SW smolts (38 days) compared to parr kept in FW, except for *slc41a3a* (Fig. 5A; c), again probably due to high variation in both groups. *cnnm4a1* expression did not change significantly between time points but remained significantly higher in smolts compared with parr after 38 days in SW, suggesting the higher level found in FW persisted in SW (Fig. 5A; g).

### Effect of photoperiodic induction of parr-smolt transformation (PST) and salinity on plasma Na^+^, Cl^-^and Mg^2+^levels

Plasma Na^+^, Cl^-^ and Mg^2+^ levels around 157.66 ± 0.49 mM, 128.56 ± 1.77 mM and 1.22 ± 0.10 mM, respectively, remained stable throughout the PST in FW. Plasma Na^+^, Cl^-^ and Mg^2+^ levels increased 1 day (166.25 ± 2.05 mM; 137.64 ± 9.27 mM; 1.61 ± 0.39 mM) and 2 days (172.03 ± 12.37 mM; 166.72 ± 2.03 mM; 1.80 ± 0.37 mM) after SW transfer in the smolt group (Fig. 5B; a-c). After 30 days in SW plasma Na^+^, Cl^-^ and Mg^2+^ levels in the smolt group returned back to 162.17 ± 1.34 mM, 125.34 ± 3.43 mM and 1.30 ± 0.06 mM, respectively. No significant difference was observed between parr and smolt groups in FW for either Na^+^, Cl^-^ and Mg^2+^ (Fig. 5B: a, b, c).

### Effect of brackish water transfer and Mg^2+^depletion on Mg^2+^transporter expression in Atlantic salmon smolts

**Fig. 6:**
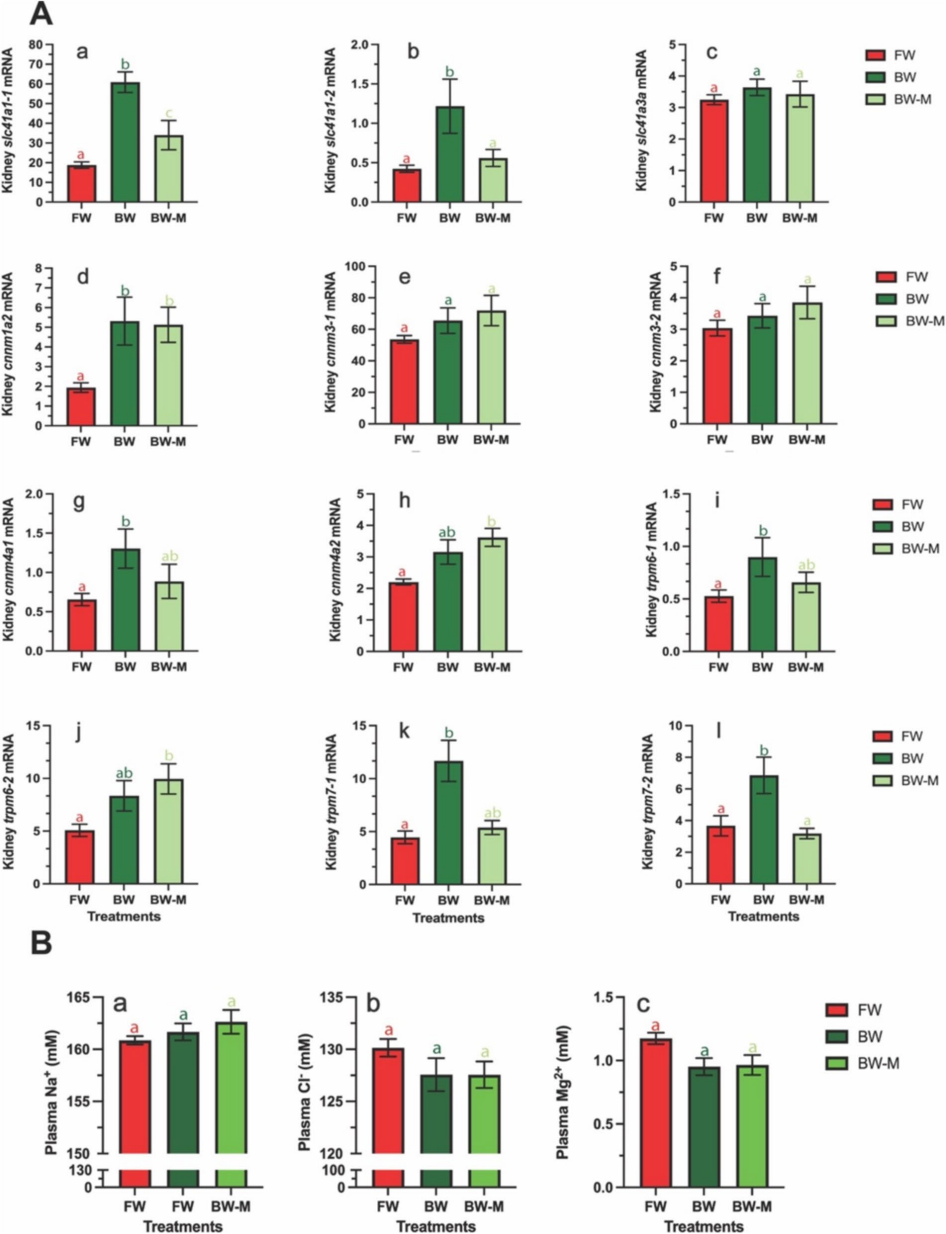
Relative gene expression levels and plasma ion concentrations in the kidney of Atlantic salmon exposed to freshwater (FW: 0.01 ppt), brackish water (BW: 12 ppt) and membrane nano-filtrated brackish water (BW-M: 12 ppt). **A** Relative expression levels of **a**: *slc41a1-1*, **b**: *slc41a1-2*, **c**: *slc41a3a*, **d**: *cnnm1a2*, **e**: *cnnm3-1*, **f**: *cnnm3-2*, **g**: *cnnm4a1*, **h**: *cnnm4a2*, **i**: *trpm6-1*, **j**: *trpm6-2*, **k**: *trpm7-1* and **l**: *trpm7-2*. **B** Ion concentrations (mM) in plasma during parr-smolt transformation in freshwater (FW), brackish water (BW) and membrane filtrated water (BW-M). **a**: sodium (Na^+^) **b**: Chloride (Cl^-^) and **c**: magnesium (Mg^2+^). Different letters denote statistically significant effects between freshwater (FW; red), brackish water (BW; dark green) and membrane nano-filtrated brackish water (BW-M; light green), reflecting the interplay of salinity between groups. Data is presented as mean ± sem (*n* = 8–10). Different letters denote statistically significant effects between freshwater (FW; red), brackish water (BW; dark green) and membrane nano-filtrated brackish water (BW-M; light green) reflecting the interplay of salinity between groups. Data is presented as mean ± sem (*n* = 8–10)

In experiment 2, smolts that had undergone PST in FW were either kept in FW, or transferred to either brackish water (BW, 12 ppt) or Mg^2+^-depleted brackish water (BW-M) for 11 weeks (Fig. 6A). We observed that *slc41a1-1*, *−2*, *cnnm4a1*, and *trpm7-2*, but also *cnnm1a2*, *trpm6-1* and *− 7 − 1* responded significantly to BW transfer (Fig. 6A; a, b, d, g, i, k, l). *cnnm4a2* and *trpm6-2* also displayed a trend towards higher expression in BW, albeit not significant (Fig. 6A; h, j). Relative expression of s*lc41a3a*,* cnnm3-1*, and c*nnm3-2* did not elicit any significant response following BW transfer (Fig. 6A; c, e, f).

Following transfer of smolts into Mg^2+^-depleted BW (BW-M) we observed that only four genes displayed a similar response compared to those in BW. The *cnnm1a2* was the only gene whose upregulation was unaltered by Mg^2+^ depletion in BW-M (Fig. 6A; d), while s*lc41a3a*,* cnnm3-1* and *− 2* displayed a similar absence of expression (Fig. 6A; c, e, f). *slc41a1-1* did display a significantly higher expression in BW-M compared to FW fish, albeit expression of *slc41a1-1* in BW-M acclimated fish were of a significant lower magnitude than the response elicited by BW transfer (Fig. 6A; a). Strikingly, the response of *slc41a1-2* and *trpm7-2* to BW was completely abolished in fish transferred in Mg^2+^-depleted BW (Fig. 6A; b, l). *cnnm4a1*,* trpm6-1*, and *trpm7-1* presented a similar trend (Fig. 6A; g, i, k). Surprisingly, *cnnm4a2* and *trpm6-2* displayed a significant higher expression in BW-M compared to FW fish, though not significantly higher than in BW (Fig. 6A; h, j).

### Effect of BW on plasma Na+, Cl- and Mg2+ levels

Plasma Na^+^, Cl^+^ and Mg^2+^ levels did not differ significantly between FW (160.86 ± 1,80 mM; 130.14 ± 3.76 mM; 1.17 ± 0.20 mM), BW (161.66 ± 2.83 mM; 127.56 ± 5.46 mM; 0.95 ± 0.23 mM) and BW-M (162.63 ± 3.96 mM; 127.55 ± 4.40 mM; 0.965 ± 0.27 mM) groups (Fig. 6B; a-c). However, plasma Mg^2+^ levels were generally lower in BW and BW-M compared to FW.

## Discussion

The SLC41A1-3, CNNM1-4 and TRPM6/7 transporter families have deep evolutionary roots and their vital role in Mg^2+^ homeostasis predates the prokaryote/eukaryote divergence [35, 67, 98]. In humans, truncations or point substitutions in most of these transporters lead to early onset of severe diseases associated with hypomagnesemia and renal and/or intestinal insufficiency as well as a suspected higher risk for neurodegenerative diseases [27, 39]. Such mutations in zebrafish have led to multiple physiological disturbances, including hypocalcemia, hypomagnesemia and development of kidney stones [36, 69, 99, 100]. The same transporters are strongly associated with Mg^2+^ regulation in the kidney of chondrichthyans, stenohaline and euryhaline teleosts, suggesting their importance for adaptations to diverse environments [30–33, 36, 101]. In salmonids the Mg^2+^ levels in urine changes from 0.49 mM in FW to 141 mM after SW acclimation, clearly indicating an important switch from net reabsorption to net secretion of Mg^2+^ in order to maintain stable plasma levels [102]. In the current study, a short-term transient rise in plasma Mg^2+^ concentration (from 1.2 mM to 1.6–1.8 mM) returns to normal physiological levels after 38 days in SW (1.3–1.4 mM). This regulation of plasma Mg^2+^ levels correlated with upregulation of *slc41a1* and *cnnm3* paralogues, and are in accordance with earlier studies concluding that these family members are leading candidates for Mg^2+^ secretion in the nephron tubules of SW teleosts and thus associated with SW acclimation [31–33]. Our results further suggest that Slc41a3, Cnnm4 and Trpm7 also may contribute to the overall removal of excess Mg^2+^ ions in SW. Our salmonid-specific data show that some of these genes are regulated in the kidney during PST (i.e., in response to photoperiod) before salinity changes. The evolution of the anadromous life-cycle is also associated with selective paralogue retention and differential regulation of paralogous gene pairs. Interestingly, the differences between pairs rely on the nature and amplitude of the trigger (seasonal changes in day length (i.e., photoperiod; PST), salinity thresholds or ion specificity). These specificities might represent evolutionary innovations in salmonids.

### Magnesium transporters — tissue distribution and regulation in vertebrates

The Atlantic salmon genome has undergone a fourth round of whole genome duplication, followed by a partial rediploidization [14]. As a consequence, the number of genes for a given family can be fairly high and the paralogous very similar in nucleotide sequence, and sometimes identical in amino acid sequence. Therefore, phylogeny and synteny analyses are recommended to infer orthologous and paralogous relationships on the genes as the published genome, do not present precise enough annotations. Our work presents the first detailed description of the salmonid members of the Slc41, Cnnm and Trpm6/7 families, and place them in the wider context of the evolution of these families across the vertebrate lineages.

**Fig. 7:**
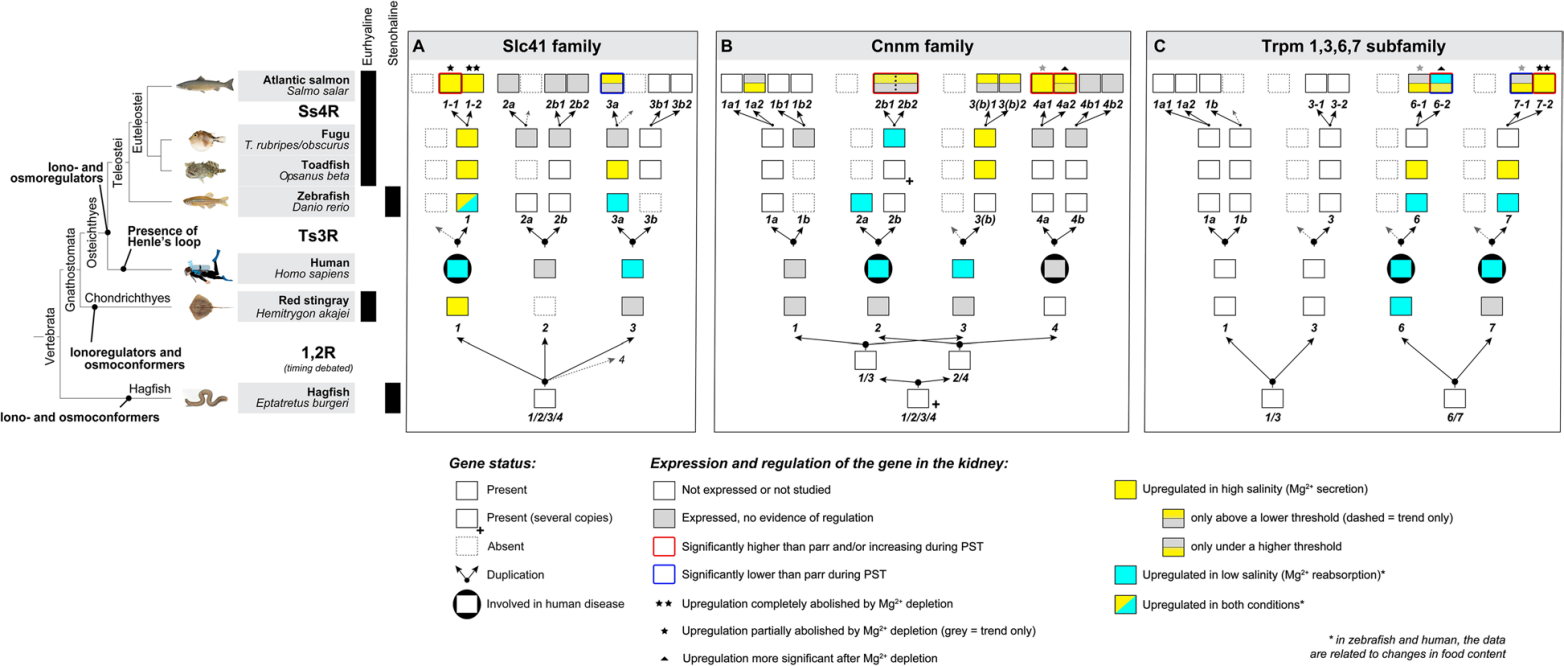
Evolution of the gene repertoire and the function of the Slc41a1-3, Cnnm1-4 and Trpm6-7 magnesium transporter families in vertebrates. This figure summarizes the presence and identity of the genes found in Atlantic salmon and compares them to other vertebrate species in which they have been functionally characterized. The occurrence of gene duplications was reconstructed through comparison of the gene repertoire from teleosts and human (Osteichthyes) with that of stingray (Chondrichthyans) and hagfish. A more complete version can be found in Fig. S1. The current knowledge on the expression of these genes in the kidney and their regulation in different environments, from our results and the literature, is illustrated (yellow and turquoise colors). For aquatic species, this information is placed in the context of the salinity tolerance of the species (euryhaline/stenohaline) as well as its ion-and osmolarity regulation methods (regulators or conformers). For Atlantic salmon, the changes associated with PST are highlighted in red or blue, and the specificity of the regulation of Mg^2+^ ions is indicated by asterisks and triangles. In the case of humans, the involvement of the gene in diseases is also represented

We have found that all six members of the Slc41a1-3 family in Atlantic salmon genome clearly has conserved PX_6_GN and P(D/A)X_4_PX_6_D motifs, indicating that key elements for selective Mg^2+^ transport are present in these proteins and areas associated with diseases was conserved. All are expressed in kidney, however only the two Ss4R *slc41a1* paralogues, *slc41a1-1* and *− 2*, as well as the remaining *slc41a3a* paralogue, are upregulated during SW acclimation (Fig. 7A). Expression of SLC41A1 and − 3 in the kidney appears to be conserved in all vertebrates, as it has been shown to be the case in a chondrichthyan, the red stingray (*Hemitrygon akajei*; [101]), and several osteichthyans including mammals and teleosts such as the fugu (*Takifugu rubripes* and *obscurus*; [31, 32]), toadfish (*Opsanus beta*; [33]), zebrafish (*Danio rerio*; [36, 37, 69]) and goldfish (*Carassius auratus*; [103]) (Fig. 7A). Interestingly, this conservation is associated with functional variations across different species. In terrestrial and FW stenohaline species, these transporters primarily play a role in reabsorption while also having critical excretory functions in euryhaline species. Functionally SLC41A1 seems to be the most important as it is being consistently regulated among vertebrates in response to Mg^2+^ changes in the environment or diet. SLC41A1 is also involved in the human disease NPHPL2 (Fig. 7A). Though Slc41a3 is expressed in kidney of red stingray, its involvement in Mg^2+^ regulation may have appeared in osteichthyans, as it is generally conserved in mammals and teleosts (Fig. 7A). Indeed, the euryhaline red stingray downregulated Slc41a1 during freshwater acclimation [101], suggesting it is involved in Mg^2+^ excretion in seawater, whilst Slc41a3 displays a much lower expression and is not regulated. Both SLC41A1 and -A3 are important for Mg^2+^ reabsorption in mammals [104]. Similarly, both Slc41a1 and − 3 family members appear to be important for Mg^2+^ regulation in teleosts, however, additional paralogues emerged after the Ts3R (Fig. 7A and S1). While only one copy of *slc41a1* has been retained, both «a» and «b» paralogues have been retained for *slc41a3*, and present signs of subfunctionalization, where *slc41a3a* appears to be the paralogue retaining the function in the kidney (Fig. 7A). In the freshwater zebrafish, *slc41a1* and *− 3a* are both important for Mg^2+^ reabsorption, and *slc41a3b* is even lost. In the kidney of euryhaline mefugu, *slc41a1* is strongly expressed and regulated, and is the key candidate to drive Mg^2+^ excretion in seawater, while *slc41a3a* is ubiquitously expressed, with the highest expression in muscle tissue, and *slc41a3b* only being detectable in the heart, brain and muscle tissues, suggesting a division of the ancestral pattern of these two paralogues [31]. In toadfish, only *slc41a1* and *-a3a* were analyzed, both of which were expressed in the kidney and upregulated in seawater ( [33], see Fig. S2 for amino acid conservation). Atlantic salmon have retained all five Slc41 members inherited from the last common ancestor of all teleosts (See also [31]), and after Ss4R, possess two copies of *slc41a1* and have lost the second copy of *slc41a3a* (Fig. 7A and S1). *slc41a1-1*,* slc41a1-2* and *slc41a3a* are all expressed in the kidney and upregulated in SW and displayed a much higher expression in the kidney than its paralogue, and as we will discuss later, are not regulated by the same environmental stimuli. While both *slc41a1* paralogues also were expressed but not regulated in gills, and undetectable in intestine, slc41a3a is expressed and appears regulated in both gills and intestine. Our results indicate an additional level of subfunctionalization between the Ss4R paralogues *slc41a1-1*,* slc41a1-2 and slc41a3a (slc41a3b1 and -b2* was undetectable; not shown) as they appear to both have different tissue distributions and environmental sensitivity.

We found twelve Cnnm paralogues in Atlantic salmon, and the residues involved in selective Mg^2+^ transport, pore functionality, as well as regions association with disease was highly conserved, strongly suggesting a retained ability to bind and transport Mg^2+^ ions. The role of the Cnnm family still remains elusive and display group- or species-specific differences but seems to have conserved functions related to Mg^2+^ transport. In mammals, CNNM2 and − 3, and to a lesser extent CNNM1 and 4, are expressed in the kidney. CNNM2, which is present in the kidney distal convoluted tubule, is thought be important for Mg^2+^ reabsorption, with sequences HOMGSMR1 and HOMG6 being associated with impaired Mg^2+^ reabsorption in the kidney and abnormal brain development [29] (Figs. 2C and 7B). CNNM3 is not associated with disease and the few studies that have addressed this family member in mammals concluded that CNNM3 is the only transporter in this family that did not generate any detectable Mg^2+^ efflux activity [105]. CNNM3 is very divergent in sequence in all vertebrates, and several amino acid substitutions concern key Mg^2+^ ion coordination sites (Fig. 2C). CNNM4 has low expression in the kidney (collecting ducts and proximal tubules, HPA) but seems critical for Mg^2+^ absorption in the intestine as shown by its involvement in Jalili syndrome [106] (Figs. 2C and 7B); CNNM1 and − 2 are also expressed in different parts of the intestinal tract, while CNNM1 is found to be important in brain and testis [107]. Interestingly, studies in mefugu and toadfish suggest an important role of Cnnm3 in kidney Mg^2+^ excretion in SW ( [32, 33], see Fig. S2 for amino acid conservation), and mefugu Cnnm3 expression in *Xenopus laevis* oocytes reportedly generated a relatively strong Mg^2+^ efflux response [32] (Fig. 7B). The ancestral state of this pattern is unclear, as red stingray has very low expression of Cnnm1, −2 and − 3 in the kidney and none of them is regulated by salinity changes [101] (Fig. 7B). The importance for Cnnm2 in the kidney is conserved among teleosts and which of the Ts3R paralogues carry this function may vary. In zebrafish, Cnnm2a is the only of the Cnnms involved in the kidney and seems to be associated with reabsorption [36, 69]. In mefugu, however, *cnnm2a* is lost and it is *cnnm2b* that is present and regulated in kidney, predominately involved in reabsorption in FW. In mefugu, *cnnm3* and *4a* are highly expressed in the kidney, followed by -*2b*, while *cnnm1b* and *− 4b* display a very low expression and *cnnm1a* is undetectable [32], indicating signs of subfunctionalization for the 1 and 4 Ts3R paralogues (Fig. 7B). Interestingly, *CNNM4* is not described to be among the strongest expressed CNNM in the mammalian kidney, while *cnnm4a* is in mefugu, potentially representing an evolutionary innovation. It is noteworthy that Cnnm4a and -b paralogues are not regulated in mefugu kidney [32]. Signs of subfunctionalization were detected for the 1 and 4 members, where *cnnm1b* and *− 4a* demonstrated broad expression across various tissues. In contrast, *cnnm1a* was strictly confined to the brain and *− 4b* mostly restricted to the brain, but also found in heart, intestine and kidney [32]. We found that salmonids retained all six members inherited from Euteleosts (*cnnm1a*, *−1b*, *−2b*, *−3*, *−4a* and *− 4b*) and retained both Ss4R paralogues for all of them (Fig. 7B and S1). Similar to findings in the mefugu [32], salmonid-specific *cnnm3* and *4a* paralogues were the most abundant in the Atlantic salmon kidney, followed by *−2b* and *− 4b*, while *− 1a* was very low (Fig. 7B). Similarly, most displayed some expression in the intestine, and *cnnm3*,* −4a*, and *− 2b* are the most prominent expressed in the gills. We observed several Ss4R paralogue-specific differences; indeed, *cnnm4a1* and − *3 − 1* clearly displayed higher expression than *cnnm4a2* and − *3 − 2* in the kidneys and the gills (Fig. 4). Similar differences were also observable for *cnnm4a1* and − *4a2* in the intestine. *cnnm4b1*,* −1a2* and *− 1b1* generally displayed higher expression in all tissues than their Ss4R paralogues, while *cnnm1b2* and *− 1a1* was not detected in the kidney (Fig. 4; *cnnm1b2*, -*1a1* not shown). While tissue distribution patterns (e.g., gills, intestine and kidney) are quite comparable between mefugu and Atlantic salmon, two euryhaline species, several major differences can be observed. First of all, salmon *cnnm1a2* seems to be the only known vertebrate *cnnm1* that is regulated by salinity, as evidenced by a significant upregulation of expression in BW. It is thus possible that it illustrates a case of neofunctionalization between *cnnm1a1* and *− 2* paralogues (Fig. 7B). Similarly, the salmon *cnnm4a1* and − *2* paralogues were the only vertebrates *cnnm4* shown to be regulated by salinity, both appearing to be significantly upregulated in SW (Fig. 7B). As both display this response but with different properties that we are going to address later, it is difficult to determine whether this is a case of neofunctionalization or neofunctionalization followed by subfunctionalization. Further, while *cnnm2b* is upregulated in FW mefugu, *cnnm2b1/2* (we could not distinguish between Ss4R paralogues for technical reasons) presents a clear trend to be upregulated in SW. Consequently, the Cnnm3 paralogues were the only transporters clearly consistent between fugu and salmon, and no sign of subfunctionalization was evident at the level of the kidney. It should be noted that some of the observed differences in expression patterns of the Cnnm family observed between salmon and fugu may reflect that tetradontiform fishes are often primarily considered to live in the marine and estuarine environment, while salmonidiformes often spend considerable time in FW, despite their anadromous life cycle. Finally, as for the SLC41A1 family, the CNNM family, through CNNM2 and − 3, appears to mainly have a reabsorbing role in mammals and zebrafish (freshwater) [106], while in euryhaline species such as mefugu and salmon, it mainly responds to higher salinities [32]. The apparent *de novo* recruitment of Cnnm1 and 4 family members in salmonids for Mg^2+^ regulation in SW might be part of a set of innovations to acclimate to SW more rapidly, or to make the regulation more intricately adaptable and thereby more plastic.

TRPM6 and − 7 are also not well characterized proteins but are believed to be Mg^2+^ channels. In mammals, TRPM6 is mostly present in the kidney and intestine [41, 108], whereas TRPM7 is ubiquitous. Both appear important for Mg^2+^ reabsorption. Many mutations in *TRPM6* and one in *TRPM7* have been found to cause HOMG1, characterized by intestinal hypomagnesemia (Figs. 3C and 7C). Expression of both genes in the kidney is conserved among vertebrates, as both are found in red stingray [101], however only *trpm6* is upregulated in freshwater red stingray indicating reabsorption function (Fig. 7C). Interestingly, teleosts also only possess 2 genes, as both genes lost one Ts3R paralogue (Fig. 7C and S1). In toadfish both *trpm6* and *− 7* are expressed in the kidney and upregulated in SW [33], indicating a potential role in excretion (Fig. 7C, see Fig. S3 for amino acid conservation). In Atlantic salmon, both genes have retained their Ss4R paralogues, and all four genes are expressed in the kidney (Fig. 4). While all four have conserved responses to salinity, clear signs of subfunctionalization, or potentially neofunctionalization can be detected. Indeed, *trpm6-1* is upregulated in response to increased salinity but only at a low level (BW but not full-strength SW), while *trpm6-2* only displays a trend towards upregulation in BW and is downregulated in full-strength SW (Fig. 7C). This makes conclusions about their relative functions complicated to propose. *trpm7-1* expression increases in BW, while *trpm7-2* strongly increase in both BW and full-strength SW. *trpm7-1* and *− 2* may therefore be involved in excretion but responding to different salinity thresholds. The function and structure of both Trpm6 and Trpm7 channels appears to be well conserved, particularly in terms of selectivity and functionality (Fig. 3C). This conservation suggests a similar function as in the mammalian kidney, where these channels play vital roles in Mg^2+^ reabsorption and overall Mg^2+^ homeostasis [45, 108, 109]. However, the activity and function of TRPM6 and TRPM7 are complex as they can form TRPM6/TRPM7 hetero-oligomers complexes that may alter the channel properties [35]. Thus, the activity of TRPM6 has been indicated to be dependent on the expression of TRPM7 [110], while mutations in TRPM6 appear to induce hypomagnesemia with secondary hypocalcemia [40].

### Differential regulation of Mg2+ transporters during PST and by environmental salinity in salmonids

Despite some Mg^2+^ transporters specifically respond to SW exposure, elevated expression of some transporters in FW smolts compared to the control (parr) suggests a developmental upregulation as a consequence of PST in response to changes in photoperiod, i.e., day length. Some Mg^2+^ transporters responded to both developmental changes and salinity changes, suggesting that some of the transporters are deployed as a preparatory measure in response to photoperiod, while some are part of an acclimation phase once they enter seawater. Overall, these intricate regulations at the kidney level contribute to avoid perturbations in plasma Mg^2+^ concentration, as observed by a very limited short-term Mg^2+^ plasma increase followed by a return to normal conditions.

**Fig. 8:**
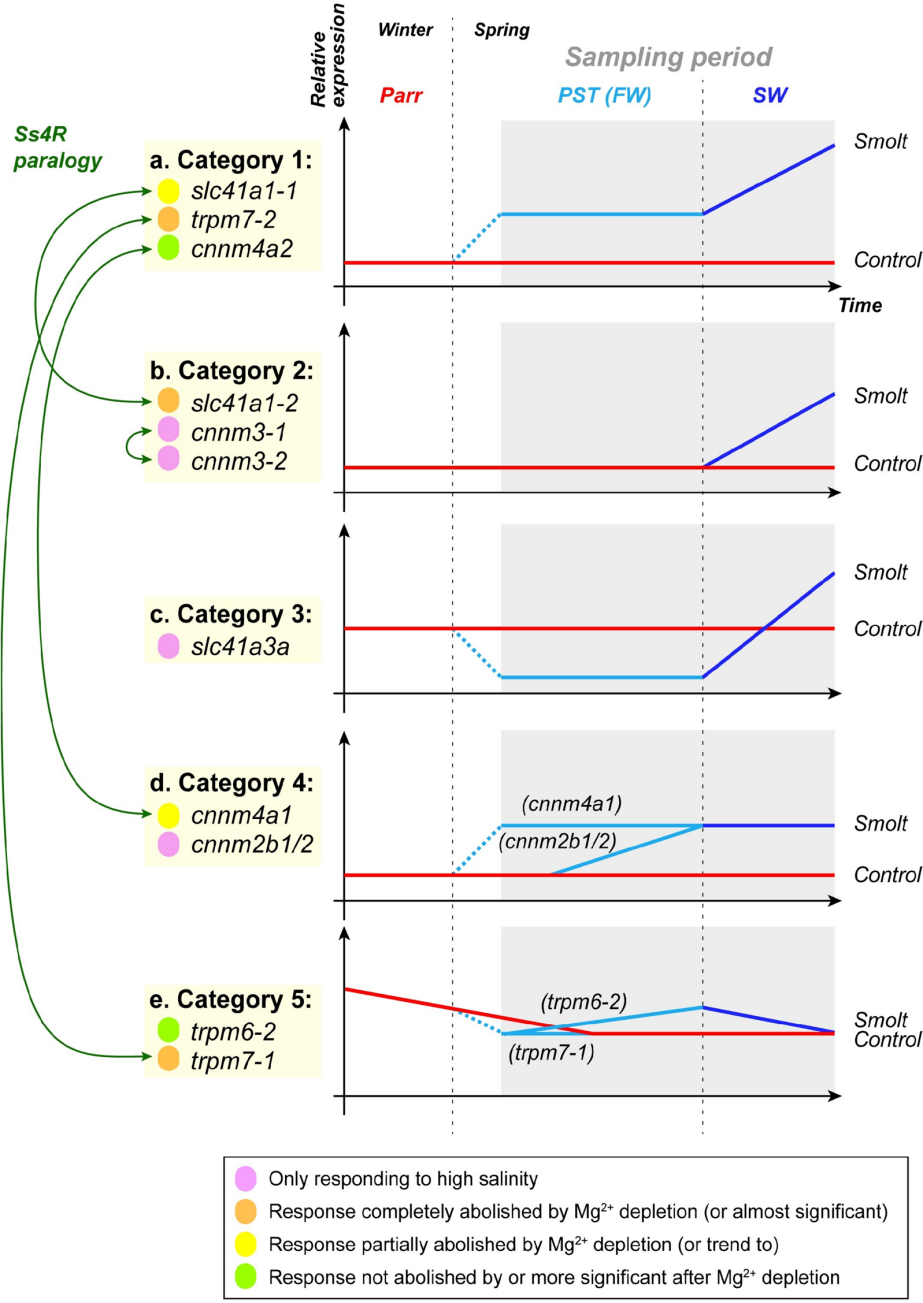
Atlantic salmon Mg^2+^transporters can be divided into five categories according to their regulation patterns, and Ss4R paralogue pairs are almost always split between different categories. **a** Category 1: upregulated by photoperiod change, then further upregulated after SW transfer. **b** Category 2: unaffected by photoperiod change, upregulated after SW transfer. **c** Category 3: downregulated by photoperiod change, then upregulated after SW transfer. **d** Category 4: upregulated by photoperiod change, but not further upregulated after SW transfer. **e** Category 5: downregulated by photoperiod change, then unchanged (*trpm7-1*) or downregulated (*trpm6-2*) after SW transfer

More concretely, we observed that the regulation of Mg^2+^ transporters in Atlantic salmon during PST and SW transfer fall into five different categories (Fig. 8). Several Mg^2+^ transporters are upregulated in response to SW (categories 1–3, Fig. 8a-c), however, among them, Mg^2+^ transporters in category 2 are exclusively upregulated in response to SW, while Mg^2+^ transporters in categories 1 and 3 also are regulated as part of PST. Mg^2+^ transporters in categories 4 and to some extent 5 (Fig. 8d, e) are not regulated in response to SW but are exclusively regulated during PST. Interestingly, Ss4R paralogue pairs are almost always split between different categories. For example, while *slc41a1-1*, *cnnm4a2* and *trpm7-2* are upregulated both during PST and after SW transfer (cat. 1, Fig. 8a), *slc41a1-2* is exclusively regulated following SW transfer (cat. 2, Fig. 8b), and *cnnm4a1* and *trpm7-1* are almost exclusively regulated during PST (cat. 4 and 5, Fig. 8d, e). Intriguingly, *trpm7-1* and *− 2* are regulated in opposite directions during PST: *trpm7-2* is upregulated (Fig. 8a), while its counterpart is slightly down-regulated (Fig. 8e). These three paralogue pairs are almost identical in amino acid sequence (Figs. 1, 2 and 3), and the difference in expression likely relies on differences in the regulatory regions of genes. Since environmental salinity and Mg^2+^ levels remain low and stable between the parr and smolt groups during PST (salinity; 0.1 ppt and Mg^2+^; 0.021 mM), we propose that the upregulation in Mg^2+^ transporters *slc41a1-1*, *cnnm4a2* and *trpm7-2*, may be governed by hormone-driven developmental signals in response to smoltification-triggered changes in day length in the smolt group. Indeed, PST is known to involve preparatory changes in many organs preparing the animal for increased salinity [111] and these changes are linked to hormonal changes [10]. Recent evidence has indicated the presence of similar adaptive switches in the kidney. Notably, we observed increases in and upregulation of leading candidates for SO_4_^2-^ excretion in the kidney (*slc26a6a1* and *slc26a1a*) during PST [24]. Together with the upregulation of the Mg^2+^ transporters in the current study, there is now a growing body of evidence suggesting that the kidney is under similar hormonal control as that previously found in the gills, which can be trigged by photoperiodic manipulation, i.e., seasonal changes in day length [10, 111, 112].

Salmonids need to tightly regulate Mg^2+^ plasma levels around 0.8–1.3 mM and successfully acclimatize to a marine environment containing 2500 fold higher Mg^2+^ levels than in FW. Thus, the increases observed in the Mg^2+^ transporters within the kidney may have an important role in preparing the fish to the rapidly increasing salinity concentrations entering the marine environment. Emerging evidence in the mammalian model suggest involvement of both insulin and growth hormone (GH) in regulating the SLC41A1 transporter [113, 114] and glucocorticoid (stimulating cortisol release) have been linked to differential regulation of TRPM6 and TRPM7 in the kidney [115]. Given the hormonal regulation observed in salmonids during PST, which includes changes in both GH and cortisol levels [10], their potential influence on Mg^2+^ transporters within the kidney serve as a fruitful field of research in the future. Thus, *slc41a1-1*, *cnnm4a2* and *trpm7-2* may all be promising candidates in Atlantic salmon. Interestingly, adult salmon returning to their natal river to reproduce show preparatory changes of ion transporters and osmoregulatory capacity, which probably are under endocrine regulation due to seasonal changes in day length [116, 117]. It is conceivable that at least some of the Mg^2+^ transporters regulated in the present study likely may display an opposite differential regulation as part preparing for FW and further acclimation to FW after entering the river. Likewise, catadromous eels may also be an interesting model for further elucidating the functional role and regulation of Mg^2+^ transporters.

### Salinity thresholds and Mg2+ specificity

We observed differences in salinity thresholds among the Mg^2+^ transporters responding to salinity changes. Indeed, *slc41a3a*, *cnnm2b1*,* −2b2*,* −3-1*, and *− 3 − 2* were significantly upregulated by a full strength seawater (SW; 33 ppt) but not by BW (12 ppt) transfer. Similarly, *cnnm4a2* only tended to respond to BW, but significantly responded to SW, while *cnnm4a1* responded significantly to both salinity levels. This suggests that some Mg^2+^ transporters require a minimum salinity threshold for their transcription to be upregulated. *trpm6-1* was significantly upregulated in BW but not SW, while *trpm6-2* only displayed a trend towards upregulation and was surprisingly downregulated in SW. *trpm7-1* appears to only be upregulated in BW and not in SW while *trpm7-2* clearly responds to both BW and SW (Fig. 7). It thus appears that paralogs not only respond differently to photoperiod or salinity in general, but also to different levels of salinity. Such regulatory patterns in response to salinity is consistent with previous findings for some transporters [23, 33], while our study is the first to show salmonid-specific paralogs within all magnesium transporter families.

We also observed variation in gene response to changes in Mg^2+^ concentrations in the water, which again could differ between Ss4R paralogues. The response of *slc41a1-1*, *slc41a1-2* and *trpm7-2* is significantly altered after Mg^2+^ reduction in BW. The response of s*lc41a1-2* and *trpm7-2* is entirely abolished, whereas the response of *slc41a1-1* appears to be dose-dependent. Moreover, while *cnnm4a1* and *trpm6-1* both significantly respond to BW, *cnnm4a2* and *trpm6-2* only respond to Mg^2+^ reduced BW, indicating they might respond to lower Mg^2+^ levels rather than salinity in general. Finally, the response of *slc41a3a*, *cnnm3-1*, and *− 2* was not significantly altered by Mg^2+^ depletion in the water. It is important to note that functional studies are needed to gain a more comprehensive understanding of these mechanisms. Nonetheless, our findings suggests that the response of *slc41a1-1* is very specific to changes in magnesium (dose dependent) while others, like s*lc41a1-2* and *trpm7-2*, need a higher magnesium threshold before they respond (respond to 49.4 and 16.55 mM magnesium but not at 9.28 mM; see Table 1). Further, the response of *slc41a3a*, *cnnm3-1*, and *− 2* is not directly affected by Mg^2+^ abundance but by overall salinity changes. For some transporters this is in line with the response observed in toad fish, which proposed that both elevated salinity and Mg^2+^ concentrations in combination was necessary to induce regulation [33]. However, our data reflects nuances in regulation of Mg^2+^ transporters, possibly equipping Atlantic salmon with a very diverse and plastic toolkit when preparing and/or acclimating to a new environment. In conclusion, our results indicate that the gene regulation for some transporters specifically respond to Mg^2+^ ions, but in-depth functional in vivo and in vitro studies on euryhaline species are required.

### Mg^2+^transporters and euryhalinity - a proposed model for the subcellular localization and the role of transporters

**Fig. 9:**
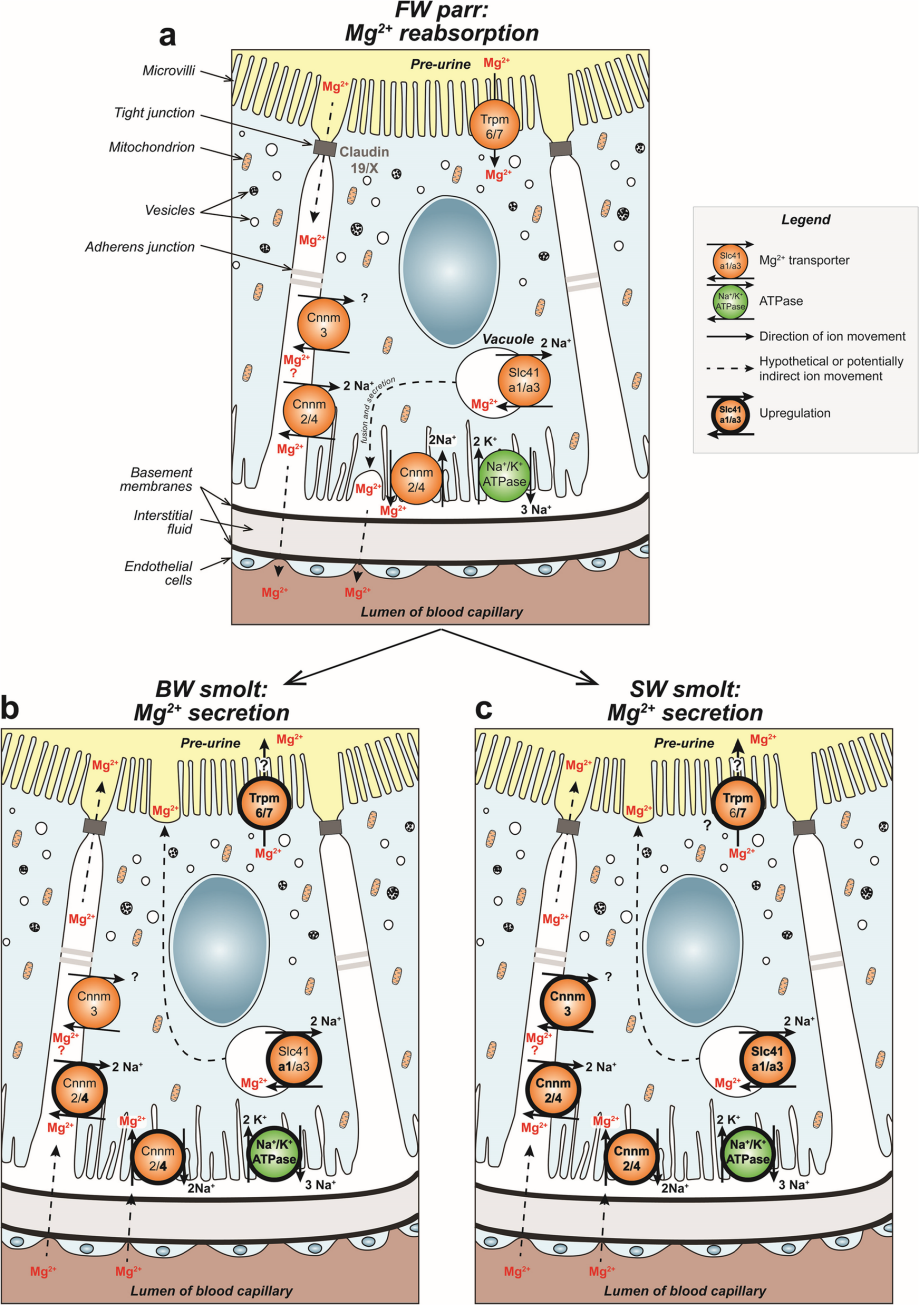
Proposed model for Mg^2+^reabsorption and secretion pathways in renal proximal tubules of Atlantic salmon (Salmo salar). The hypothesized models are based on works from mammalian [34, 108, 110] and teleost [30–32, 37, 45] models in conjunction with the expression profiles for salmon *slc41a1* and *− 3*, *cnnm2*, *−3* and *− 4*, and *trpm6* and *− 7* in the current study. The basolateral Na+/K+ - ATPase pump establishes an electrochemical gradient, that is the driving force required to both reabsorb and secrete Mg^2+^ through the transcellular pathway against the concentration gradient in FW, BW and SW environments. **a** FW parr. We propose that in FW, the NKA pump provides the foundation for inward flow of Mg^2+^ through the apically located channels Trpm6/7 (found at the apical membrane in the distal convoluted tubule (DCT) in mammals), followed by Mg^2+^ extrusion through the basolateral located Cnnm4 and Cnnm2 transporters. Cnnm3 may also be involved, though their function is unknown in FW fish. Additionally, we propose that the Slc41a1/a3 transporters, as suggested in pufferfish, are responsible for accumulating Mg^2+^ from the cytosol into vacuoles, which after exocytosis, lead to basolateral Mg^2+^ secretion. Intercellular Mg^2+^ will further be transported into the blood. A passive paracellular route likely also exists through permeable channels formed by tight junction claudin proteins (Cldn). In mammals, Cldn16, Cldn19 and Cldn14 are known to be involved at the level of the Thick Ascending limb of Henle’s loop (TAL; [45]), however only Cldn19 has been found in the fish kidney and more research is needed to characterize the homo- or heterodimeric interaction between Cldn19 and other unknown claudin isoforms. **b** BW smolt. As Mg^2+^ concentration increases, fish need to switch from net reabsorption to net secretion of Mg^2+^. As the Mg^2+^ concentration gradient across the cells is opposite to the one in FW, we propose that the transcellular pathway responsible for Mg^2+^ secretion is the exact opposite of the one in FW. Basolateral located CNNM4 transporters drive an inwards flow of Mg^2+^. While functional studies are required to verify it role during salinity acclimation, we propose that Trpm6/7 may enable secretion of Mg^2+^ at the apical membrane into the pre-urine. The upregulation of both Cnnm4 and Trpm6/7 indicates a need for increased transport. Apical secretion would also further be supported by the apical exocytosis of Slc41a1 (upregulated as well)/a3-positive Mg^2+^-enriched vacuoles (Na^+^/Mg^2+^ exchanger). In parallel to this, we suggest that Cnnm2 and Cnnm4, and possibly Cnnm3, extrude Mg^2+^ into the lateral intercellular space in exchange for Na^+^. Then Mg^2+^ likely exits through the claudin-associated paracellular pathway. **c** SW smolt. While we think the mechanisms in SW are largely similar to the ones in place in BW, a second set of transporters are upregulated in response to higher Mg^2+^ concentrations in SW. Slc41a3 upregulation would enhance vacuole accumulation and apical secretion of Mg^2+^, while Cnnm2 and 3 upregulation would enhance Mg^2+^ extrusion to the intercellular space, feeding the paracellular transport pathway

The Slc41a and Cnnm transporters in fishes have further been characterized at the cellular and subcellular levels by in situ hybridization and immunohistochemistry analyzes. Slc41a1, -a3, cnnm2, −3 and − 4, all suspected to be Na^+^/Mg^2+^ exchangers and were all shown to be expressed proximal tubules of the kidney [31, 69, 118]. However, their subcellular localizations appear to differ. Slc41a1 and Slc41a3 SW pufferfish were found on the membrane of subapical vesicles, whereas the Cnnm2 and − 3 were detected on lateral membranes and the Cnnm4 was found more basolateral ([31, 32], Fig. 9). Therefore, Slc41a1 and Cnnm3, proposed as the main regulators of magnesium in SW kidney, would both secrete Mg^2+^ but through different mechanisms at different subcellular localizations, e.g., Slc41a1 likely secretes Mg^2+^ through exocytosis via the apical membrane, while Cnnm3 appears to be important in lateral Mg^2+^ extrusion (Fig. 9).

In the FW goldfish the kidney can change its transport direction when given a high Mg^2+^ diet, shifting from net reabsorption to net secretion [103]. This suggest that Slc41a1, and potentially Slc41a3, may have dual roles in responding to salinity levels from dietary and/or environmental sources, adapting specifically to meet the animal’s requirement within its habitat (FW vs. SW and terrestrial vs. aquatic). This scenario is also proposed for euryhaline fishes like mefugu [31]. Thus, we propose that in Atlantic salmon, Slc41a1-1, -a1-2, and -a3a, are involved in intracellular accumulation of Mg^2+^ in vacuoles, and that these vacuoles are either routed to the basal side in FW to contribute to Mg^2+^ reabsorption (Fig. 9A), or to the apical membrane in BW and SW to enable secretion of Mg^2+^ (Fig. 9B, C). Regarding the differential regulation of paralogues, we propose that *slc41a1-1* is first upregulated during PST as a preparative measure, enabling an immediate and sufficient response to salinity changes, followed by a further increase in *slc41a1-1* expression when the animal does encounter higher salinity. Moreover, *slc41a1-1* may further be aided by the upregulation of *slc41a1-2* and *slc41a3a*, which is required to remove sustained high Mg^2+^ concentrations as they enter the marine environment. We hypothesize that these may also be associated with apical-directed vacuolar transport, a mechanism that was first proposed by earlier physiological studies in marine fish [26, 30] and later supported by molecular studies in pufferfish [31].

Similar to the SLC family members, Cnnm3 is thought to have a secretory function for Mg^2+^ in euryhaline fugu and toadfish. If so, the Slc41 paralogues would be central in the transcellular pathway while Cnnm3 would more support an additional paracellular secretion [32]. The increase of *cnnm3-1* and *− 2* after SW exposure in Atlantic salmon is consistent with these interpretations. Previous findings suggest that Cnnm3 is important for lateral Mg^2+^ extrusion. We therefore propose that Cnnm3-1 and − 2 always extrude Mg^2+^ on the lateral membrane, however, it should be noted that the transcellular pathway has a different orientation in FW vs. BW and SW (Fig. 9A versus B, C), which contribute to either reabsorption or secretion of Mg^2+^.

CNNM2 and − 4 are highly expressed in renal distal convoluted tubule cells at the basolateral membrane, and they are thought to be the most likely basolateral transporters responsible for mediating Mg^2+^ transport [118, 119]. CNNM2 is important for reabsorption in mammals, euryhaline fugu and stingray, however upregulation of Cnnm2b1/2 in Atlantic salmon during PST, indicate it might be part of the preparatory changes prior to entering higher salinity environment. Similar to the role of Cnnm3-1 and − 2, we propose that Cnnm2b1/2 may be involved in extruding Mg^2+^ on the lateral membrane and contribute to the paracellular pathway (Fig. 9). CNNM4 is expressed in the kidney of humans and euryhaline fugu but have never been shown to be regulated by food intake or salinity changes. We therefore suggest that the observed upregulation of the *cnnm4a* paralogues in response to PST and SW transfer may be an evolutionary innovation. Furthermore, *cnnm4a1* seems to be only involved in preparatory changes during PST, whereas *cnnm4a2* also is upregulated in response to elevated salinity. Based on our current understanding, it is probable that these transporters have dual functions in euryhaline fish, allowing them to adapt and switch the transport direction of Mg^2+^ ions in accordance to the animals surrounding environment (salinity levels in food and/or environment). Such compensatory mechanisms are likely to be particularly robust in euryhaline fish, such as Atlantic salmon, which experience substantial changes in salinity during their life cycle. Thus, it is plausible that these transporters facilitate the removal of excess Mg^2+^ from the extracellular space into the intracellular space in high salinity environments where it can further be transported through the apical and lateral transporters Slc41a1/Slc41a3 and Cnnm3, respectively (Fig. 9). Future functional studies on all these transporters are required to verify our hypothesized cellular model in fish, and if a directional shift in Mg^2+^ transport (reabsorption to secretion) is possible as Mg^2+^ concentrations in and around the nephron cells significantly change during SW transitions.

In human and zebrafish, TRPM6 and 7 are thought to be involved in renal Mg^2+^ reabsorption. In mammals, these Mg^2+^ - channel proteins mainly contribute to reabsorption, presumably by facilitating apical Mg^2+^ entry in the distal tubules [28, 45, 108, 120]. Both *trpm6* and *− 7* expression are upregulated toadfish exposed to SW [33]. Similarly, we observed a significant increase in the salmonid-specific *trpm7-2* paralogue during PST and SW acclimation, while *trpm6-1* and *trpm7-1* only responded to BW, and *trpm6-2* only respond to BW-M and only slightly increased during PST. Our findings together with Hansen and co-authors [33] clearly suggest a role in Mg^2+^ secretion in SW, at least for *trpm7.* It is important to note that TRPM6 and TRPM7 have been referred to as the TRPM6/7 complexes, which enables constant supply of Mg^2+^ regardless of the physiological state. Furthermore, TRPM6 has been proposed to have a modulating effect on the function of TRPM7, whilst the channel activity of TRPM7 is tightly controlled by cytosolic Mg^2+^ and Mg*ATP [44]. In the current study the transcriptional pattern of the *trpm7-1* in Atlantic salmon, appears to be non-responsive in Mg^2+^ depleted BW (9.28 mM) while responding to higher Mg^2+^ levels in both BW (16.6 mM) and SW (49.4 mM). We therefore suggest that these transporters are involved in Mg^2+^ reabsorption in FW (Fig. 9A) and may have the capacity to facilitate transport in the reverse direction, potentially supporting Mg^2+^ excretion in BW and SW acclimated fish (Fig. 9B, C). The differential response to Mg^2+^ concentrations in the water, also suggest that these channels may be upregulated in response to environmental Mg^2+^ levels. Nevertheless, it is important to note that this hypothesis requires validation through dedicated functional and immunolocalization studies at the protein and cellular level, particularly in euryhaline species. Even in mammals the exact Mg^2+^ regulatory mechanisms are not fully understood, and despite identifying several important Mg^2+^ transporters and regulators the last decade, our knowledge of their physiological relevance is still limited [28].

### Magnesium transporters and kidney disease

Mutations in SLC41A1 have been shown to be associated with NPHPL2, with an early onset of degeneration of kidney structure, including periglomerular fibrosis, tubular ectasia, tubular basement membrane disruption, and tubulointerstitial infiltrations [38]. *slc41a1* knock-down in zebrafish also results in kidney cysts [38]. HOMGSMR1 and HOMG6 are caused by mutations in CNNM2 and are characterized by low plasma magnesium and abnormally high renal excretion of magnesium associated with neurological disorders [43, 69]. Similarly, morpholino knockdown of zebrafish *cnnm2a* and *-b* both resulted in decreased total body Mg^2+^ levels, and cnnm*2a* KO also caused renal cysts [69]. TRPM6 and 7 are involved in HOMG1, characterized by hypomagnesemia with secondary hypocalcemia and is associated with calcinosis (stones) in several organs, including kidneys [41]. Mutations in CLDN16 and − 19 also cause hypomagnesemia, associated with hypercalciuria and nephrocalcinosis (HOMG3 and 5 [121]), . Similar physiological disturbances as that observed in mammals have been observed in Atlantic salmon where development of kidney stones (termed nephrocalcinosis) with severe consequences for the animal [122, 123]. Yet, the molecular and physiopathological mechanisms underlying nephrocalcinosis remains unclear [4]. Our results indicate that paralogues of the genes involved in kidney Mg^2+^ regulation share key structural and functional domains and residues in the human version. Thus, the conserved function and disease related sites indicate that Atlantic salmon could be used as model organism in both functional and disease related research.

### Conclusion and perspectives

Magnesium homeostasis is a universally central requirement at a cellular and systemic level, yet the molecular bases of Mg^2+^ transport in vertebrates have only been identified in the last decade. A growing number of studies, including the present one, have begun to reveal the broad conservation of the Mg^2+^ transporter genes and their regulation in mammals and teleosts species. Teleosts, with their ability to occupy diverse environments that vary widely in Mg^2+^ content, serve as valuable models for studying Mg^2+^ regulation and physiopathology related to perturbations in Mg^2+^ homeostasis. While zebrafish are a stenohaline freshwater species, many teleost species, including salmonids, have evolved the capacity to transition between different salinities. Therefore, studying the evolution of Mg^2+^ transporters/channels and their regulation, can highlight important conserved mechanisms and provide insights into adaptive innovations through the evolution of vertebrates. In particular, additional rounds of duplication in teleost, especially in salmonids, are a source of neo-and subfunctionalization events that can be associated with evolutionary innovations. To conclude, beyond describing the biology of Atlantic salmon, we believe these results further help establish salmon as a relevant biological model to understand endogenous and exogenous regulation of ion homeostasis. Through analysis of recently duplicated paralogous genes we may dissect how genome evolution can provide tools for new evolutionary adaptions. Functional studies will be the next step on this path.

## Supplementary Information

Additional file 1: Fig. S1. Evolutionary scenarios proposed to account for the magnesium transporter gene repertoire in extant vertebrate groups. For a set of representative vertebrate species, genes from the SLC41 (A), CNNM (B) and TRPM1/3/6/7 (C) present in the genome were displayed as filled squares. The color code used indicates the orthology relationships inferred from the phylogenetic and synteny analyses, with black squares indicating a suggested pre-2WGD common ancestor. We have decided to represent the teleost- and salmonid-specific paralogues with the same color as their pre-Ts3R or -Ss4R WGD orthologues, for simplicity. Chromosomal linkage is represented by a shared gray line between several genes. Proposed gene losses and gains along the evolution of the different groups were then mapped on the phylogenetic tree connecting the species. The putative gene repertoire in the common ancestor after a WGD is also presented in a box in the proximity of the corresponding event on the tree.

Additional file 2: Fig. S2. Relative gene expression levels and plasma ion concentrations in the kidney of Atlantic salmon during parr-smolt transformation (FW; 0.1 ppt) and seawater (SW; 35 ppt) acclimation. Relative expression levels in the kidney of Atlantic salmon during parr-smolt transformation (PST; 0.01 ppt) and seawater (SW; 35 ppt) acclimation. Complete figure containing all the studied genes (not all are included in the main text). a: cnnm1b1, b: cnnm1a2, c: slc41a2a, d: slc41a2b1, e: slc41a2b2 and f: slc41a2b2X3. Different letters denote statistically significant difference between time points within the parr (red) and smolt (FW: light blue, SW: dark blue) groups. An asterisk (*) indicates significant differences between parr and smolt groups tat a given time point. Note that the parr group (control) remains in freshwater throughout the experimental period. Data is presented as mean ± sem (*n* = 8-10).

Additional file 3: Fig. S3. Relative gene expression levels and plasma ion concentrations in the kidney of Atlantic salmon exposed to freshwater, brackish water (BW) acclimation. Relative expression levels in the kidney of Atlantic salmon exposed to freshwater, brackish water (BW) acclimation. Complete figure containing all the studied genes (not all are included in the main text). a: slc41a2a, b: slc41a2b1, c: slc41a2b2, d: cnnm2b-1/2, e: cnnm1b and f: slc41a2aX3. Different letters indicate statistically significant differences between freshwater (FW; red), brackish water (BW; dark green) and membrane nano-filtrated brackish water (BW-M; light green). Data is presented as mean ± sem (*n *= 8-10).

Additional file 4: Fig. S4. Evolutionary conservation of aminoacid residues across vertebrates in the SLC41 (A) and CNNM (B) families. Complete figure containing all the studied species (not all are included in the main text).

Additional file 5: Fig. S5. Evolutionary conservation of amino acid residues across vertebrates in the TRPM6/7 family. Complete figure containing all the studied species (not all are included in the main text).

## Data Availability

All data generated or analyzed during this study and the supplementary information are available in the submitted version. The Salmo salar genome DNA and protein sequencing data have been used from the most recent genome release Ssal_v3.1/GCA_905237065.2.
