# Supplementary figures and images for: Differential regulation of magnesium transporters Slc41, Cnnm and Trpm6-7 in the kidney of salmonids may represent evolutionary adaptations to high salinity environments
Source: BMC Genomics. 2024 Nov 29;25:1156. doi: 10.1186/s12864-024-11055-x (PMC11605958; doi:10.1186/s12864-024-11055-x)

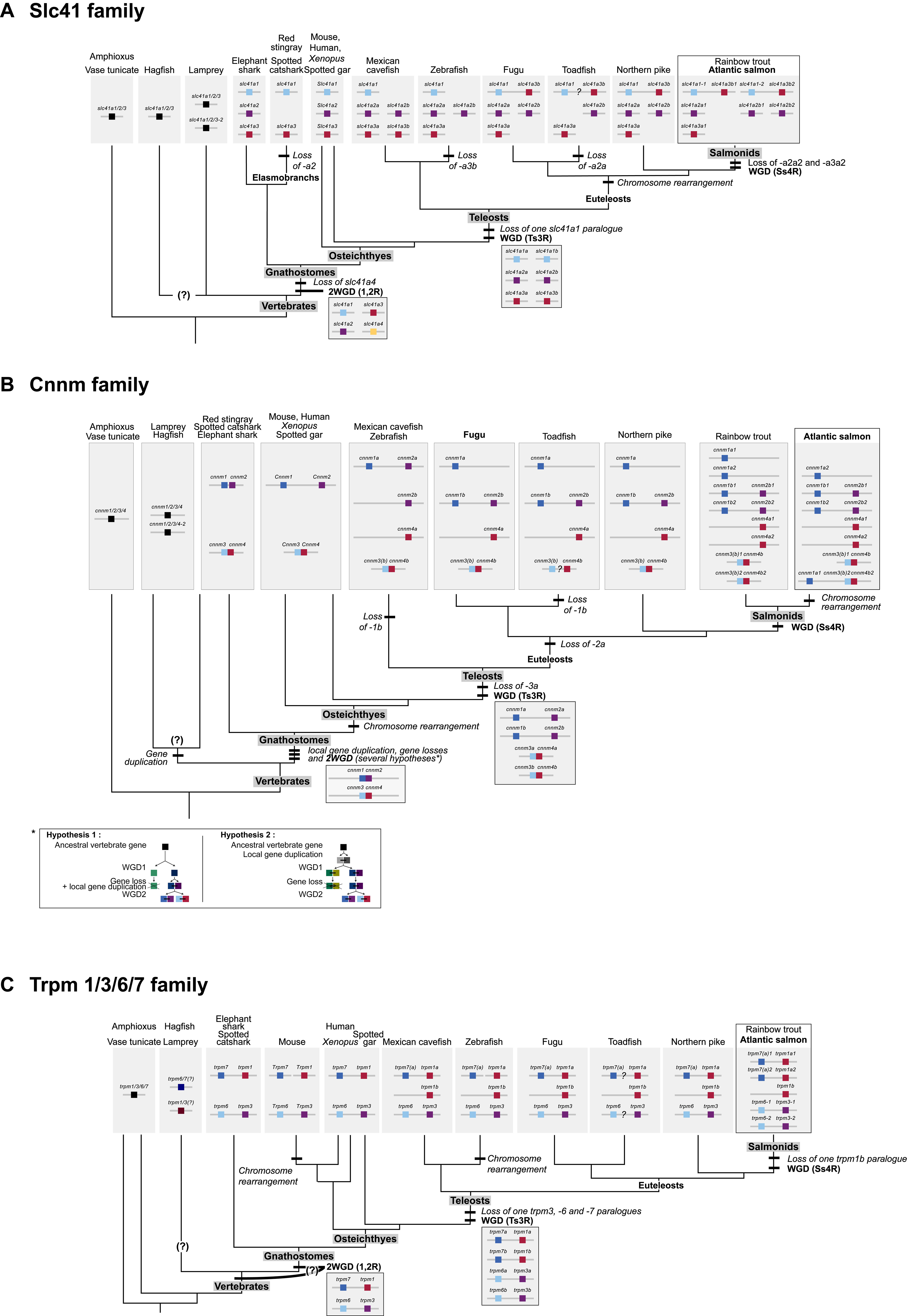

Supplement: Supplementary file 1 — Additional file 1: Fig. S1. Evolutionary scenarios proposed to account for the magnesium transporter gene repertoire in extant vertebrate groups. For a set of representative vertebrate species, genes from the SLC41 (A), CNNM (B) and TRPM1/3/6/7 (C) present in the genome were displayed as filled squares. The color code used indicates the orthology relationships inferred from the phylogenetic and synteny analyses, with black squares indicating a suggested pre-2WGD common ancestor. We have decided to represent the teleost- and salmonid-specific paralogues with the same color as their pre-Ts3R or -Ss4R WGD orthologues, for simplicity. Chromosomal linkage is represented by a shared gray line between several genes. Proposed gene losses and gains along the evolution of the different groups were then mapped on the phylogenetic tree connecting the species. The putative gene repertoire in the common ancestor after a WGD is also presented in a box in the proximity of the corresponding event on the tree. [file 12864_2024_11055_MOESM1_ESM.jpg]

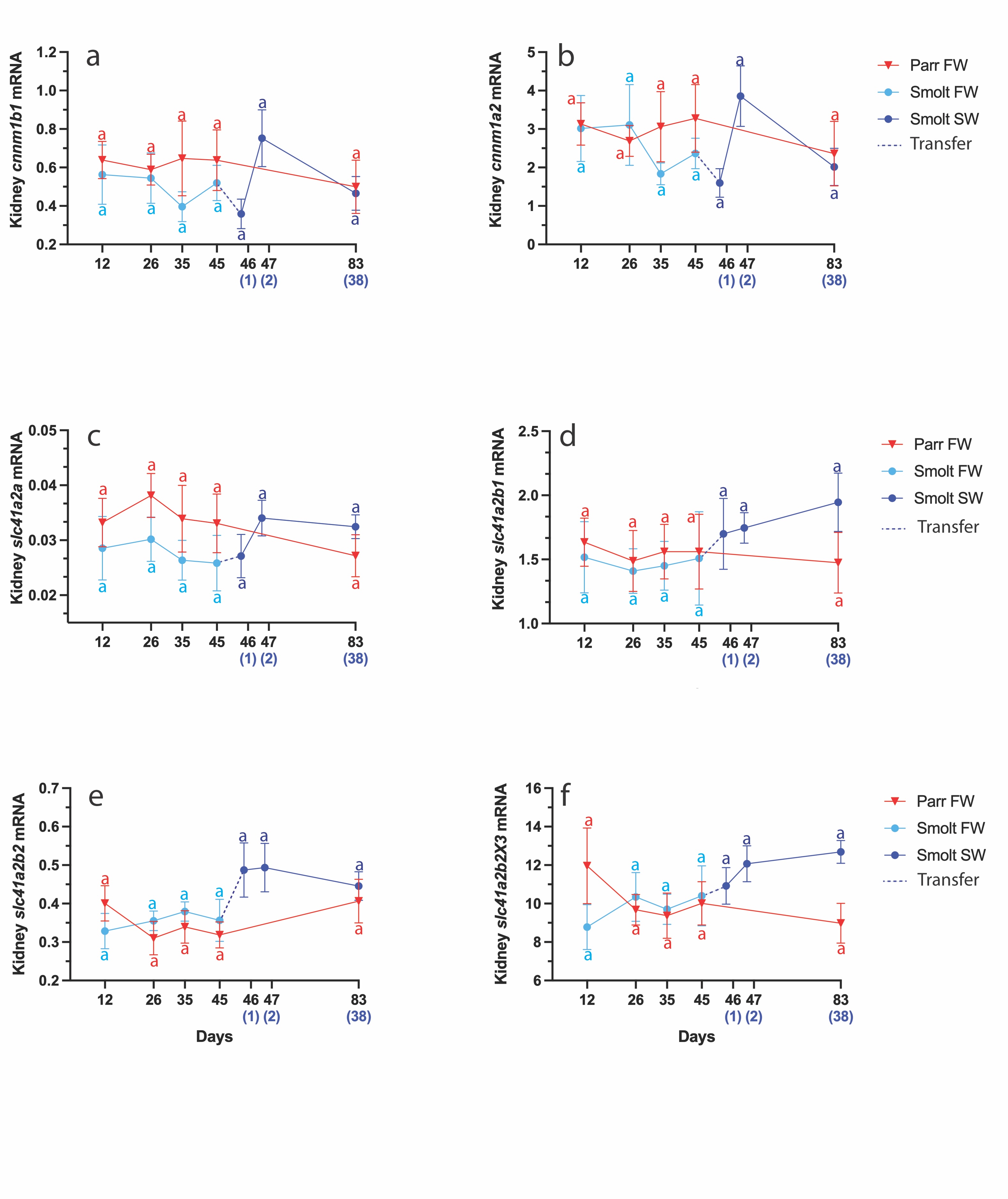

Supplement: Supplementary file 2 — Additional file 2: Fig. S2. Relative gene expression levels and plasma ion concentrations in the kidney of Atlantic salmon during parr-smolt transformation (FW; 0.1 ppt) and seawater (SW; 35 ppt) acclimation. Relative expression levels in the kidney of Atlantic salmon during parr-smolt transformation (PST; 0.01 ppt) and seawater (SW; 35 ppt) acclimation. Complete figure containing all the studied genes (not all are included in the main text). a: cnnm1b1, b: cnnm1a2, c: slc41a2a, d: slc41a2b1, e: slc41a2b2 and f: slc41a2b2X3. Different letters denote statistically significant difference between time points within the parr (red) and smolt (FW: light blue, SW: dark blue) groups. An asterisk (*) indicates significant differences between parr and smolt groups tat a given time point. Note that the parr group (control) remains in freshwater throughout the experimental period. Data is presented as mean ± sem (n = 8-10). [file 12864_2024_11055_MOESM2_ESM.jpg]

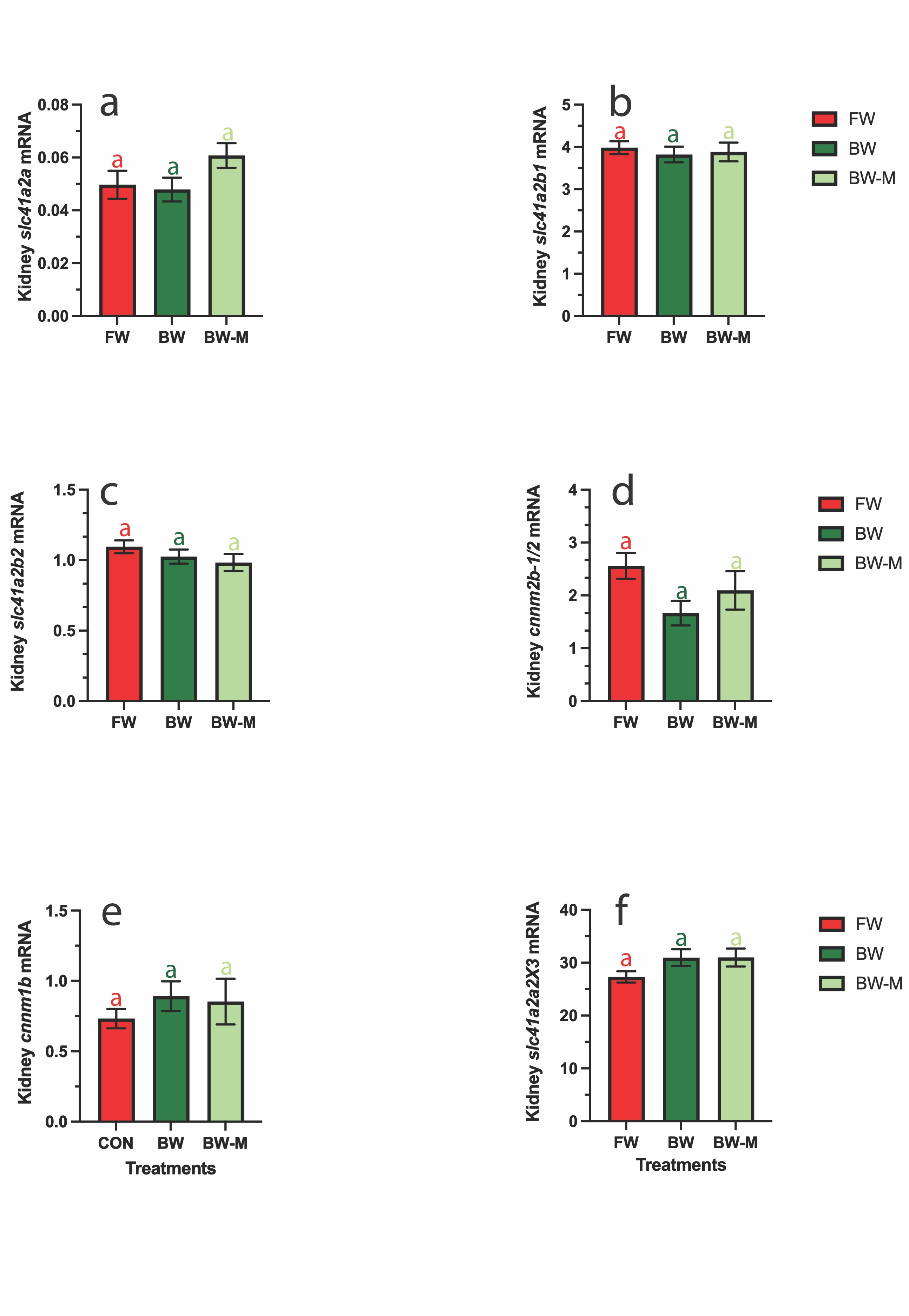

Supplement: Supplementary file 3 — Additional file 3: Fig. S3. Relative gene expression levels and plasma ion concentrations in the kidney of Atlantic salmon exposed to freshwater, brackish water (BW) acclimation. Relative expression levels in the kidney of Atlantic salmon exposed to freshwater, brackish water (BW) acclimation. Complete figure containing all the studied genes (not all are included in the main text). a: slc41a2a, b: slc41a2b1, c: slc41a2b2, d: cnnm2b-1/2, e: cnnm1b and f: slc41a2aX3. Different letters indicate statistically significant differences between freshwater (FW; red), brackish water (BW; dark green) and membrane nano-filtrated brackish water (BW-M; light green). Data is presented as mean ± sem (n = 8-10). [file 12864_2024_11055_MOESM3_ESM.jpg]

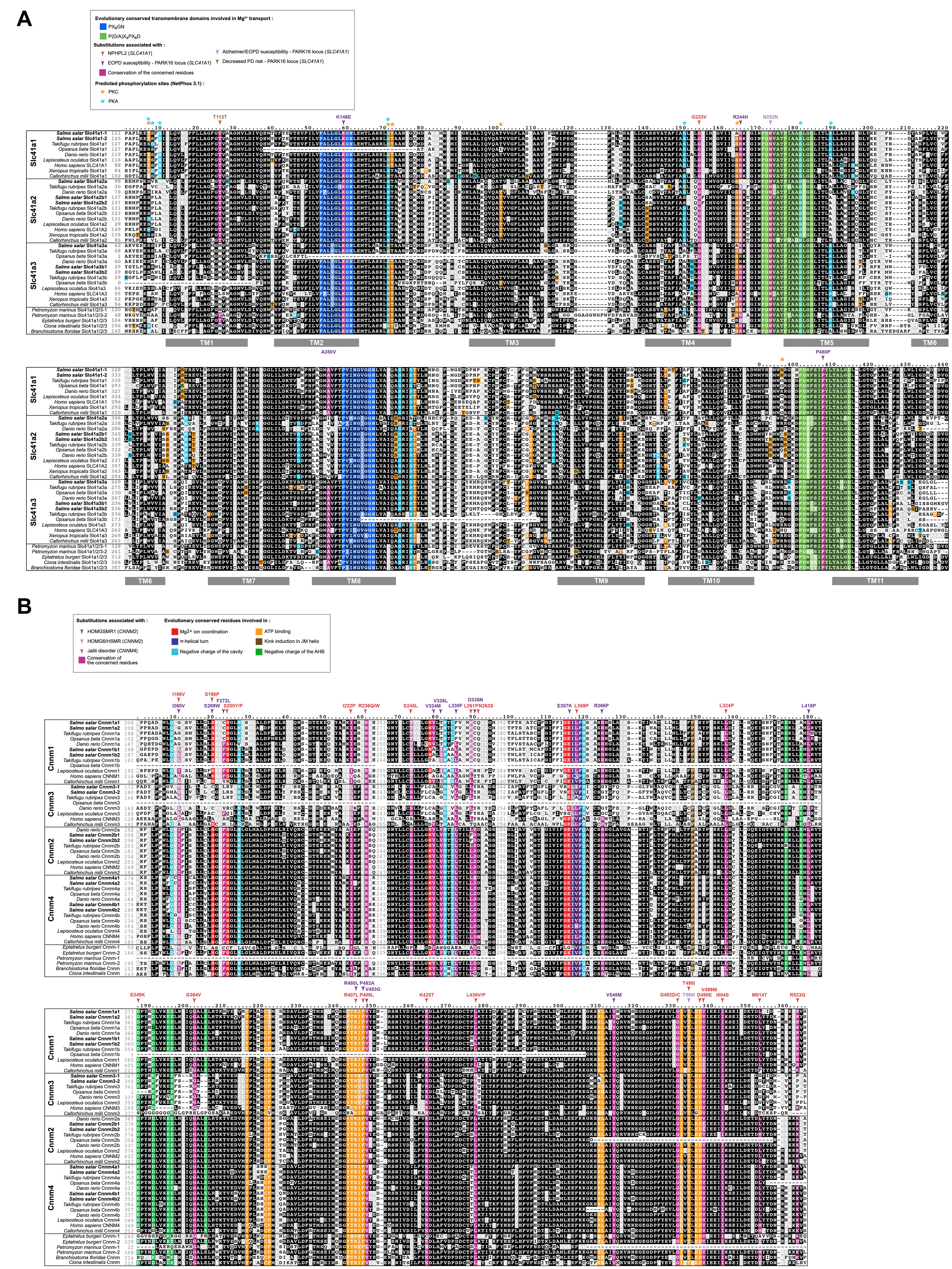

Supplement: Supplementary file 4 — Additional file 4: Fig. S4. Evolutionary conservation of aminoacid residues across vertebrates in the SLC41 (A) and CNNM (B) families. Complete figure containing all the studied species (not all are included in the main text). [file 12864_2024_11055_MOESM4_ESM.jpg]

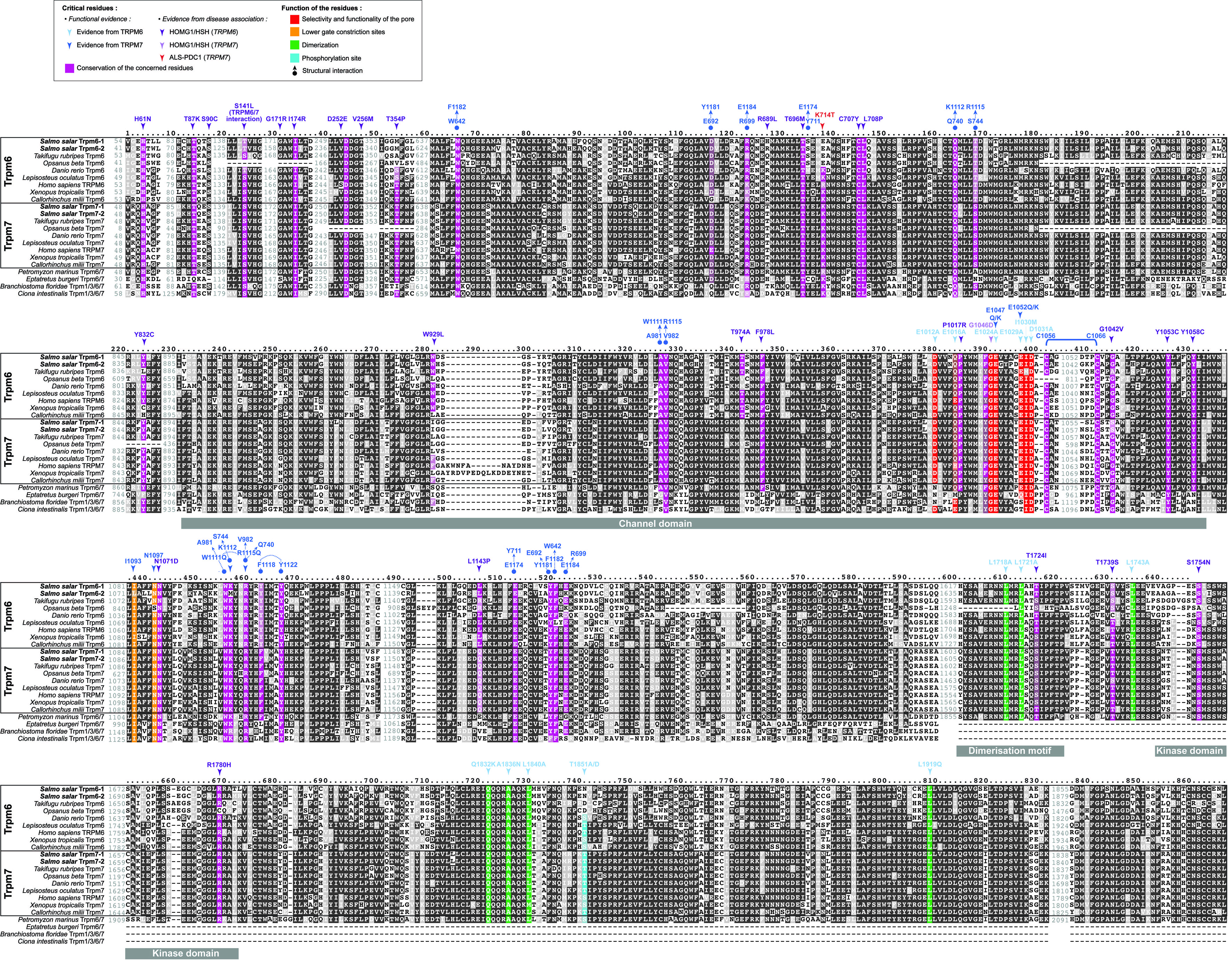

Supplement: Supplementary file 5 — Additional file 5: Fig. S5. Evolutionary conservation of amino acid residues across vertebrates in the TRPM6/7 family. Complete figure containing all the studied species (not all are included in the main text). [file 12864_2024_11055_MOESM5_ESM.jpg]
